# The CKS1/CKS2 Proteostasis Axis Is Crucial to Maintain Hematopoietic Stem Cell Function

**DOI:** 10.1097/HS9.0000000000000853

**Published:** 2023-02-28

**Authors:** William Grey, Samantha Atkinson, Beatrice Rix, Pedro Casado, Linda Ariza-McNaughton, Cathy Hawley, Miriam L. Sopoena, Katherine S. Bridge, David Kent, Pedro R. Cutillas, Dominique Bonnet

**Affiliations:** 1 York Biomedical Research Institute, Department of Biology, University of York, United Kingdom; 2 Hematopoietic Stem Cell Laboratory, The Francis Crick Institute, London, United Kingdom; 3 Cell Signalling and Proteomics Group, Centre for Genomics and Computational Biology, Bart’s Cancer Institute, London, United Kingdom; 4 Bioinformatics Core, The Francis Crick Institute, London, United Kingdom

## Abstract

Long-term hematopoietic stem cells are rare, highly quiescent stem cells of the hematopoietic system with life-long self-renewal potential and the ability to transplant and reconstitute the entire hematopoietic system of conditioned recipients. Most of our understanding of these rare cells has relied on cell surface identification, epigenetic, and transcriptomic analyses. Our knowledge of protein synthesis, folding, modification, and degradation—broadly termed protein homeostasis or “proteostasis”—in these cells is still in its infancy, with very little known about how the functional state of the proteome is maintained in hematopoietic stem cells. We investigated the requirement of the small phospho-binding adaptor proteins, the cyclin-dependent kinase subunits (CKS1 and CKS2), for maintaining ordered hematopoiesis and long-term hematopoietic stem cell reconstitution. CKS1 and CKS2 are best known for their roles in p27 degradation and cell cycle regulation, and by studying the transcriptome and proteome of *Cks1*^*−/−*^ and *Cks2*^*−/−*^ mice, we demonstrate regulation of key signaling pathways that govern hematopoietic stem cell biology including AKT, FOXO1, and NFκB, together balancing protein homeostasis and restraining reactive oxygen species to ensure healthy hematopoietic stem cell function.

## INTRODUCTION

The hematopoietic system is a prototypical stem cell hierarchy characterized by quiescent long-term hematopoietic stem cells (HSCs) residing at the top, with more differentiated multipotent, lineage restricted progenitors carrying out the bulk of hematopoiesis during homeostasis.^1^ Although rarely in cell cycle during steady-state, HSCs remain essential to replenish the hematopoietic system after injury or infection.^2–4^

Although HSC function is very well described at the cellular level, the underlying molecular mechanisms that confer high potency and life-long self-renewal remains an open question. Landmark studies have revealed key biomarkers^5,6^ and molecular mechanisms discriminating true HSCs from primitive progenitors,^7–9^ but most of this work has relied on transcriptomic profiling. The state of the proteome has been less well reported, and indeed recent reports have demonstrated an uncoupling of the transcriptome and proteome in HSCs resulting in poor overlap between some key regulators.^10^ What is also clear from new studies, is that HSCs require highly regulated rates of protein synthesis and a decline in these quality control measures inhibits HSC self-renewal.^11,12^ Specifically, genetic deletion of ubiquitin proteosome components *c-Cbl*,^13^ *Itch*,^14^ *Fbw7*,^15^ *Mdm2*,^16^ and *Skp2*^17^ all result in altered HSC phenotypes associates with self-renewal and proliferation, demonstrating the need for precisely regulated protein degradation in HSCs. We and others have reported the importance of protein homeostasis—so called proteostasis—in HSCs,^7,18–21^ and here we report the requirement of the cyclin-dependent kinase subunits, CKS1 and CKS2, for balancing protein signaling networks in HSCs.

The CKS proteins were originally identified as subunits of cyclin–CDK complexes, important for managing substrate specificity and multisite phosphorylation of CDK targets.^22–24^ More recently, both CKS1 and CKS2 were identified to have CDK-independent roles associated with ubiquitin ligase complexes involving Skp2 and Cdc20.^25–27^ Through these interactions, CKS1 and CKS2 balance proteostasis throughout the cell cycle, ensuring correct G_0_/G_1_ transition,^25^ chromatin separation,^27,28^ and DNA repair.^25,29,30^ Previously, it was reported that CKS1 is required for the maintenance of HSC quiescence.^31^ We reported more recently the regulation of the key hematopoietic transcription factor Mll1 by CKS1 and CKS2,^26^ with downstream consequences on Wnt signaling. Here we investigated the role of the CKS1/CKS2 axis in HSCs, demonstrating that balancing this axis is critical to maintain proteostasis through multiple key signaling pathways fundamental for HSC transplantation ability.

## MATERIALS AND METHODS

### LT-HSC transplantation

Immunophenotypic CD45.2–positive LT-HSCs (Live, Lineage marker negative, cKit^+^Sca1^+^CD48^-^CD150^+^CD34^−^CD135^−^) from *Cks1/2*^*−/−*^ mice were sorted on a BD ARIAFusion into PBS + 1% fetal bovine serum (FBS). Four hundred LT-HSCs were injected intravenously in lethally irradiated (6.5 Gy × 2 doses) wild type (WT) CD45.1 mice alongside 10^6^ CD45.1 whole bone marrow cells. Primary recipient mice were bled from the tail every 4 weeks to assess total chimerism (CD45.1 versus CD45.2), myeloid cell chimerism (CD11b and Gr-1), B cell chimerism (B220), and T cell chimerism (CD4 and CD8). After 24 weeks, primary recipient mice were sacrificed, and bone marrow and spleens were harvested for analysis by flow cytometry. CD45.2-positive LT-HSCs (400) were sorted and transplanted into lethally irradiated secondary recipient WT Bl6 CD45.1 mice with 10^6^ CD45.1 whole bone marrow cells. These mice were analyzed by tail bleeds as per primary recipients for 24 weeks and mice were sacrificed, and bone marrow and spleen were harvested for analysis by flow cytometry.

### Colony-forming unit assay

Colony-forming units were assessed using cytokine-supplemented methylcellulose (StemCell Technologies M3434-GF). 10^4^ lineage marker negative cells were seeded in cytokine supplemented methylcellulose and colony-forming units were scored after 7 days as per the manufacturer’s instructions.

### Flow cytometry

Flow cytometry analyses were performed using a BD Fortessa LSRII flow cytometer (BD Biosciences). Cells were prepared from peripheral blood, bone marrow (through flushing by centrifugation), and spleen (through crushing), lysed in red blood cell lysis buffer (155 mM NH_4_Cl, 12 mM NaHCO_3_, 0.1 mM EDTA) for 5 minutes at 4°C and washed in PBS + 1% FBS before staining. Bone marrow and spleen cells were lineage depleted using the stem cell technologies hematopoietic progenitor isolation kit as per the manufacturer’s instructions (StemCell technologies #19856) before staining. Cells were stained with cell surface antibodies (key resources table) in PBS + 1% FBS for 1 hour at 4°C, washed 3 times in PBS + 1% FBS and resuspended in PBS + 1% FBS + 4′,6-diamidino-2-phenylindole (0.1 μg/mL) before analysis by flow cytometry. For intracellular flow cytometry, cells were first stained with cell surface antibodies and fixable live dead dye (either Zombie UV Biolegend or Sytox Green Life Technologies) followed by fixation in 1.6% formaldehyde (Sigma) at room temperature for 10 minutes in the dark and permeabilized in 1 mL Perm buffer III (BD biosciences) on ice for 30 minutes in the dark. Cells were washed 3 times in PBS + 1% FBS and incubated with intracellular antibodies for 4 hours at 4°C. Stained cells were washed three times in PBS + 1% FBS and analyzed by flow cytometry.

### NADP/NADPH assays

Total nictoinamide adenine dinucleotide phosphate (NADP/H) and NADPH were measured using the NADP/NADPH colorimetric assay kit (Abcam). Equal number of sorted LSK cells (Live, lineage marker negative, cKit^+^Sca1^+^) were washed 3 times in ice-cold PBS and lysed in NADP/NADPH extraction buffer by performing 2 freeze/thaw cycles (20 minutes on dry ice followed by 10 minutes at room temperature). Lysates were centrifuged at 13,000*g* for 10 minutes at 4°C and the supernatant was retained. Lysate supernatant was split in half, with one half remaining on ice and the other half incubated at 60°C for 30 minutes to remove NADP^+^. Total NADP/H (NADPt) and NADPH only lysates were run in 96-well plates with freshly made standards as per the manufacturers’ instructions. NADP/NADPH ratio was calculated as (NADPt-NADPH)/NADPH.

### Intracellular reactive oxygen species staining

CellROX deep red (Life Technologies) was used to assay intracellular reactive oxygen species (ROS). LSK cells were sorted and incubated with 5 μM CellROX deep red and 50 μM verapamil for 1 hour at 37°C, 5% CO_2_. Cells were washed with PBS + 1% FBS + 50 μM verapamil + 4′,6-diamidino-2-phenylindole (0.1 μg/mL) and ROS were assayed by flow cytometry.

### Statistical analysis

Results shown are ±SEM unless otherwise indicated. To compare *Cks1*^*−/−*^ and *Cks2*^*−/−*^ versus WT control in all in vitro and in vivo experiments, an analysis of variance was calculated. All repeat samples presented are from biological replicates of distinct mice. Correlation analyses were carried out using the “performance analytics” and “corrplot” packages in R. Pathway analysis and enrichment was run through MetaCore (genego.com). Further analysis of proteomes and transcriptomes are detailed in the Suppl. materials.

### Publicly available datasets

Chromatin immunoprecipitation sequencing data were obtained from chip-atlast.org and downloaded from the target gene section with ±1k from TSS and average MACS binding score >1 across all datasets. CKS1 and CKS2 paired gene and protein expression in LT-HSCs and multipotent progenitors (MPPs) (Suppl. Figure S1A) was obtained from Zaro et al.^10^

## RESULTS

### Loss of CKS1 or CKS2 results in expansion of the hematopoietic stem and progenitor cell compartment

**Figure 1.**
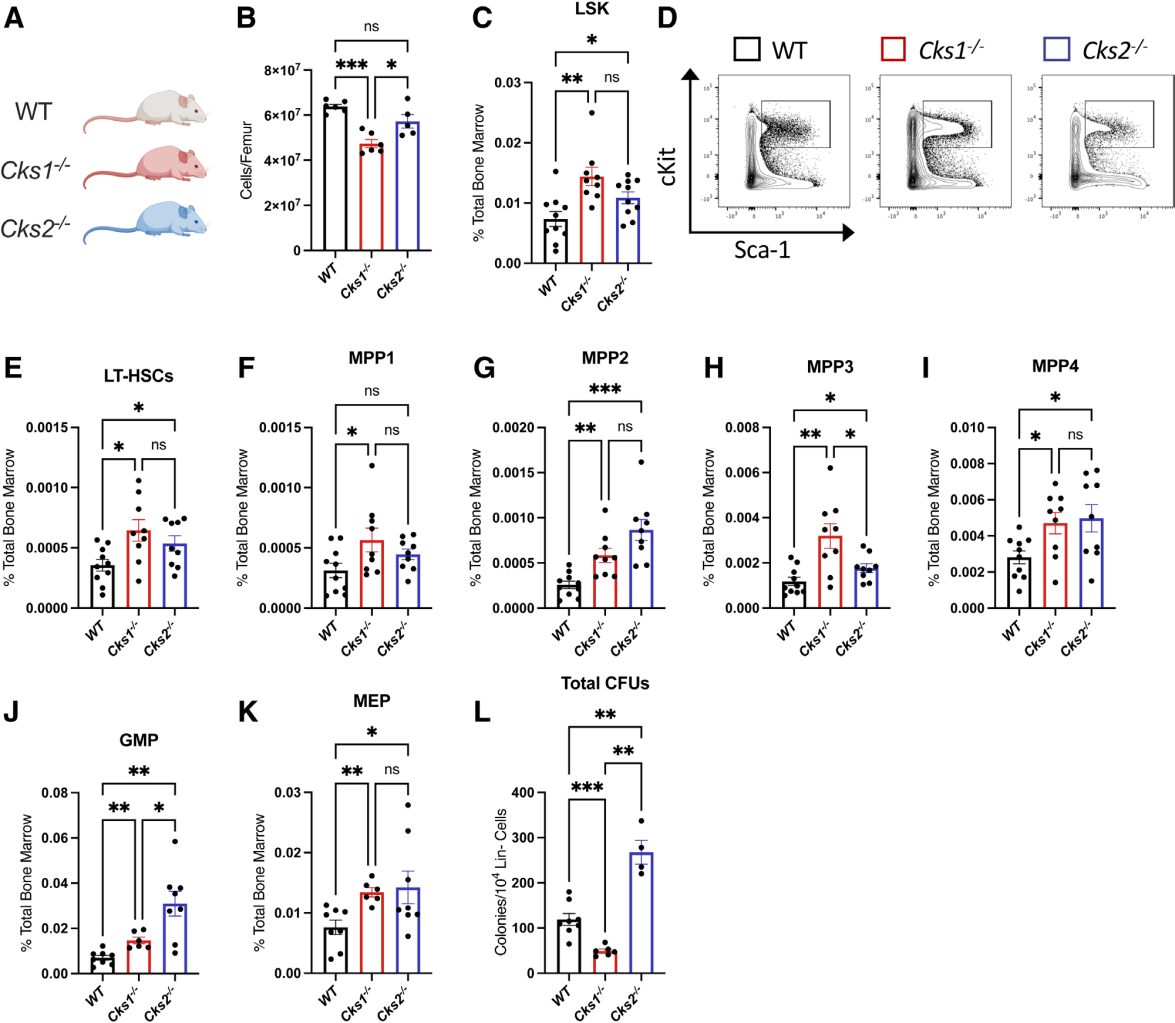
**Hematopoietic profile of *Cks1*^−^/^−^ and *Cks2*^−^/^−^ mice.** (A) Color coding for mouse models (white = WT, red = *Cks1*^*−/−*^, blue = *Cks2*^*−/−*^). (B) Total cell number per femur. (C) Percentage LSK cells of total bone marrow. (D) Example flow plot for LSK cells. Percentage (E) LT-HSCs, (F) MPP1, (G) MPP2, (H) MPP3, (I) MPP4, (J) GMP, (K) MEP of total bone marrow. (L) Number of colony-forming units per 10^4^ lineage negative cells seeded. For all graphs, a one-way ANOVA was used to calculate significance of differences. *P < 0.05, **P < 0.005, ***P < 0.0005. ANOVA = analysis of variance; GMP = granulocyte myeloid progenitor; LT-HSC = long-term hematopoietic stem cell; LSK = lineage marker negative, Sca1^+^, cKit^+^; MEP = megakaryocyte erythroid progenitor; MPP = multipotent progenitor; WT = wild type.

CKS1 and CKS2 vary at both the RNA and protein level in the most primitive HSC and early progenitor populations (Suppl. Figure S1A). *Cks1*^*−/−*^ mice are smaller than WT and *Cks2*^*−/−*^ littermates^25^ and consequently have fewer cells per femur (Figure 1A–B). We carried out immunophenotypic analysis of the bone marrow in *Cks1*^*−/−*^ and *Cks2*^*−/−*^ mice and found an expansion of primitive hematopoietic populations, including increased proportion and estimated absolute number of long-term HSCs (LT-HSCs), MPPs, and committed progenitors (Suppl. Figure S1B–G, Figure 1C–K).

The higher proportion of progenitors in *Cks2*^*−/−*^ mice compared with WT controls was matched by higher colony-forming activity of bone marrow cells, but interestingly *Cks1*^*−/−*^ bone marrow cells showed the opposite phenotype, with fewer colonies formed than WT controls (Figure 1L, Suppl. Figure S2A–E). These results were consistent across all types of colonies formed except erythroid colonies, where *Cks2*^*−/−*^ bone marrow cells have lower blast forming unit erythroid (BFU-E) potential compared with WT controls (Suppl. Figure 2D). CKS1 and CKS2 are critical regulators of cell cycle regulation, and upon inspection of the cell cycle profile in HSPC subsets, we found similar profiles to those previously published in other tissues, with *Cks1*^*−/−*^ cells enriched in G_1_ phase at the expense of SG_2_M phases and *Cks2*^*−/−*^ cells enriched in SG_2_M phases at the expense of G_1_ phase (Suppl. Figure 2F–J). These data demonstrate an expansion of immunophenotypic hematopoietic stem and progenitor cells in the bone marrow of *Cks1*^*−/−*^ and *Cks2*^*−/−*^ mice, interestingly without direct correlation to colony-forming ability in *Cks1*^*−/−*^ mice.

### *Cks1* ^*−/−*^ and *Cks2* ^*−/−*^ HSCs have poor transplantation and repopulation potential

**Figure 2.**
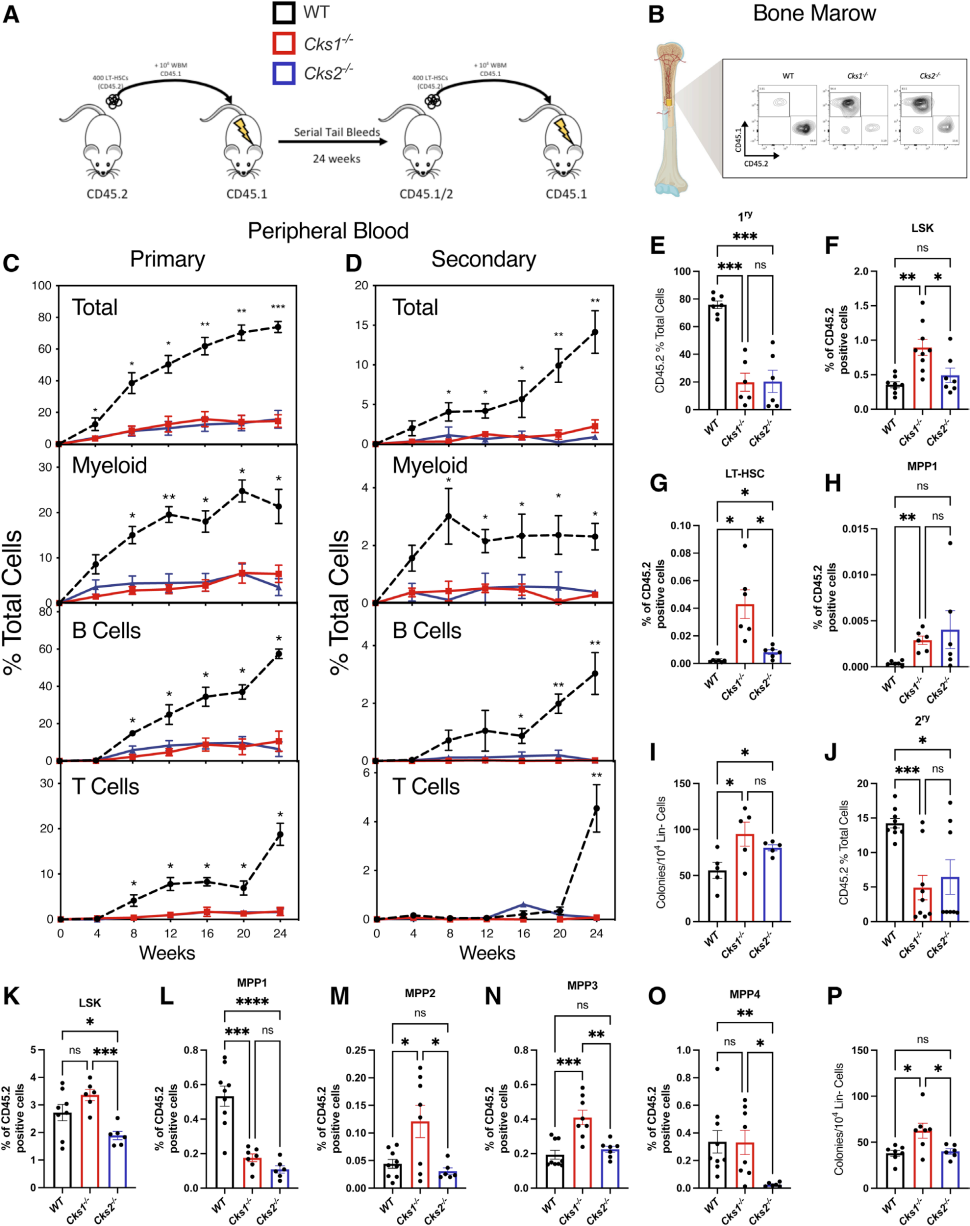
**Serial transplantation of LT-HSCs from WT, *Cks1*^−^/^−^ and *Cks2*^−^/^−^ mice.** (A) Schematic of transplantation in primary and secondary recipient mice (white/black line = WT, red = *Cks1*^*−/−*^, blue = *Cks2*^*−/−*^). (B) Example flow plot for CD45.1 vs CD45.2 staining to demonstrate chimerism differences between WT, *Cks1*^*−/−*^ and *Cks2*^*−/−*^ donors after 24 wk in a primary recipient. Percentage of total, myeloid, B and T donor derived cells in the peripheral blood of (C) primary and (D) secondary recipient mice. (E) Percentage donor-derived CD45.2 cells of total CD45-positive cells in the bone marrow of primary recipient mice. Percentage (F) LSK, (G) LT-HSC, and (H) MPP1 cells of total CD45.2 donor-derived cells in primary recipients. (I) Number of colony-forming units per 10^4^ lineage negative CD45.2-positive cells seeded from primary recipients. (J) Percentage donor-derived CD45.2 cells of total CD45-positive cells in the bone marrow of secondary recipient mice. Percentage (K) LSK, (L) MPP1, (M) MPP2, (N) MPP3, and (O) MPP4 cells of total CD45.2 donor-derived cells in secondary recipients. (P) Number of colony-forming units per 10^4^ lineage negative CD45.2-positive cells seeded from secondary recipients. For all graphs, a 1-way ANOVA was used to calculate significance of differences. *P < 0.05, **P < 0.005, ***P < 0.0005. ****P < 0.0001. ANOVA = analysis of variance; LT-HSC = long-term hematopoietic stem cell; LSK = lineage marker negative, Sca1+, cKit+; MPP = multipotent progenitor; WT = wild type.

To assess the functionality of the expanded LT-HSC population in *Cks1*^*−/−*^ and *Cks2*^*−/−*^ mice (both CD45.2 positive), we sorted 400 immunophenotypic LT-HSCs (LSK:CD48^-^CD150^+^CD34^−^Flt3^−^; Suppl. Figure S1B) and transplanted them into lethally irradiated recipient mice with whole bone marrow supporting cells from WT CD45.1–positive mice and monitored recipients for 24 weeks to test primary reconstitution ability, followed by resorting and transplanting in secondary recipients for a further 24 weeks to test long-term HSC functionality (Figure 2A–B). Both *Cks1*^*−/−*^ and *Cks2*^*−/−*^ LT-HSCs failed to fully reconstitute the peripheral blood of primary or secondary recipients (Figure 2C), with significantly reduced donor derived cells in peripheral blood across all blood lineages (myeloid, B and T cells; Figure 2C–D).

Immunophenotypic analysis of the bone marrow and spleen of primary recipients confirmed the results obtained from the peripheral blood (Figure 2E and Suppl. Figure S3A, respectively). Interestingly, while both knockout grafts were reduced, the proportion of LSK, LT-HSCs, and MPP1 were significantly enriched in *Cks1*^*−/−*^ grafts compared with WT controls and a trend toward enrichment in *Cks2*^*−/−*^ grafts was observed (Figure 2F–H). Despite the enrichment of these primitive cell subsets, MPPs2-4, common myeloid progenitors (CMPs), granulocyte myeloid progenitors (GMPs), and megakaryocyte erythroid progenitors MEPs) were comparable between *Cks1*^*−/−*^, *Cks2*^*−/−*^, and WT controls (Suppl. Figure S3B–G). Additionally, lympho-myeloid primed progenitors (LMPPs) were reduced in *Cks1*^*−/−*^ grafts (Suppl. Figure S3H) and common lymphoid progenitors (CLPs) were increased in *Cks2*^*−/−*^ grafts (Suppl. Figure S3I) compared with WT controls. The enrichment in stem and progenitor cells in *Cks1*^*−/−*^ and to a lesser degree in *Cks2*^*−/−*^ grafts resulted in higher colony-forming ability than WT controls (Figure 2I, Suppl. Figure S3J). This result directly contrasts with the lower clonogenic activity of primary *Cks1*^*−/−*^ bone marrow cells (Figure 1L).

Secondary recipients showed a similar profile to primary recipients, with significantly lower chimerism in peripheral blood throughout (Figure 2D) and significantly lower bone marrow (Figure 2J) and spleen (Suppl. Figure S3K) chimerism after 24 weeks compared with WT controls. Interestingly, in secondary recipients, the LSK compartment was no longer increased in *Cks1*^*−/−*^ grafts and significantly reduced in *Cks2*^*−/−*^ grafts compared with WT controls (Figure 2K). Additionally, there was a switch between primary and secondary grafts, with equal proportions of LT-HSCs (Suppl. Figure S3L) and reduced MPP1 cells (Figure 2L) in *Cks1*^*−/−*^ and *Cks2*^*−/−*^ secondary grafts compared with WT controls. Instead, *Cks1*^*−/−*^ and *Cks2*^*−/−*^ cells were enriched in mid-MPP stages, (MPP2s, MPP3s, and MPP4s (Figure 2M–O). Lineage committed progenitors, with the exception of MEPs, were comparable with WT controls (Suppl. Figure S3M–Q). This resulted in only *Cks1*^*−/−*^ secondary engrafted cells having a higher colony-forming ability compared with both *Cks2*^*−/−*^ and WT controls (Figure 2P, Suppl. Figure S3R). To test whether these phenotypes were a result of inefficient homing of LT-HSCs, we tested the ability of LSK cells to home to the bone marrow after 16 hours. *Cks1*^*−/−*^ LSK cells showed comparable homing to WT controls and *Cks2*^*−/−*^ cells had a slight reduction in homing, but cells were still detected in the bone marrow of recipient mice (Suppl. Figure 3S). To further confirm the phenotype we observed in *Cks2*^*−/−*^ donors, we injected LT-HSCs intra-bone to WT recipients from WT, *Cks1*^*−/−,*^ and *Cks2*^*−/−*^ donors (Suppl. Figure 3T). Chimerism of intra-bone injected recipient mice (Suppl. Figure 3T) was significantly reduced in *Cks1*^*−/−*^ and *Cks2*^*−/−*^ donors, similar to intravenous injected LT-HSCs (Figure 2C).

These data suggest that, both *Cks1*^*−/−*^ and *Cks2*^*−/−*^ LT-HSCs have a reduced ability to reconstitute the hematopoietic system upon transplantation.

### Proteomic analysis reveals key hematopoietic signaling pathways altered in *Cks1* ^*−/−*^ *and Cks2* ^*−/−*^ HSPCs

**Figure 3.**
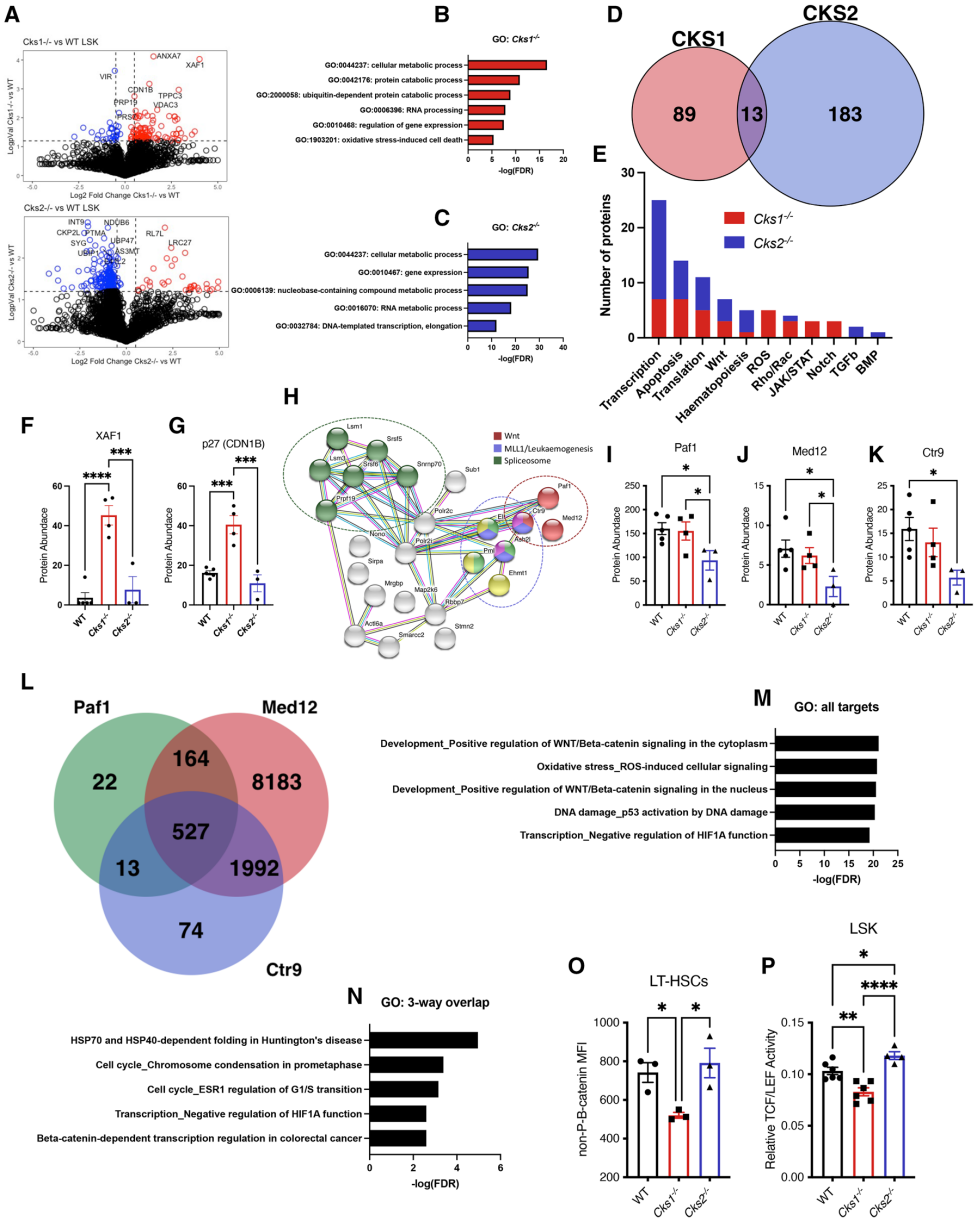
**Proteomic analysis of *Cks1*^−^/^−^ and *Cks2*^−^/^−^ hematopoietic stem and progenitor cells.** (A) Volcano plots for differentially abundant proteins measured by mass spectrometry between *Cks1/2*^*−/−*^ LSK cells and WT controls (red circles are significantly upregulated proteins, blue circles are significantly down regulated proteins). Gene ontology analysis of differentially abundant proteins in (B) *Cks1*^*−/−*^ and (C) *Cks2*^*−/−*^ LSK cells vs WT controls. (D) Venn diagram illustrating overlap of proteins differentially abundant between *Cks1/2*^*−/−*^ LSK cells and WT controls. (E) Annotated biological process for differentially abundant proteins in *Cks1*^*−/−*^ (red) and *Cks2*^*−/−*^ (blue) LSK cells. Protein abundance of known CKS1 interactors (F) XAF1 and (G) p27 from mass spectrometry analyses. (H) String network analysis of transcription associated proteins differentially abundant in either *Cks1*^*−/−*^ or *Cks2*^*−/−*^ LSK cells compared with WT controls. Protein abundance of (I) Paf1, (J) Med12, and (K) Ctr9 from mass spectrometry analyses. (L) Venn diagram depicting overlap of genes bound by Paf1, Med12, and Ctr9 from Chip-atlas.org. Gene ontology analysis of (M) all genes bound by either Paf1, Med12, or Ctr9 and (N) genes bound by all 3 proteins (Paf1, Med12, and Ctr9). (O) MFI of nonphosphorylated-β-catenin in LT-HSCs from WT and *Cks1/2*^*−/−*^ mice. (P) Relative TCF/LEF activity in LSK cells transfected with TOPFlash constructs and cultured for 48 h. For all graphs, a 1-way ANOVA was used to calculate significance of differences. *P < 0.05, **P < 0.005, ***P < 0.0005. ****P < 0.0001. ANOVA = analysis of variance; LT-HSC = long-term hematopoietic stem cell; LSK = lineage marker negative, Sca1^+^, cKit^+^; MFI = mean fluorescence intensity; TCF/LEF = T cell factor/lymphoid enhancer factor; WT = wild type.

CKS1 and CKS2 balance protein phosphorylation and ubiquitin-mediated degradation through interactions with CDKs, SCF^SKP2^, and APC^CDC20^.^21–26^ To investigate the underlying biology of hematopoietic phenotypes in *Cks1*^*−/−*^ and *Cks2*^*−/−*^ mice, we carried out proteomic evaluation of stem and early progenitor populations (LSK; Figure 3A, Suppl. Figure S4A). *Cks1*^*−/−*^ cells had more significantly upregulated proteins and *Cks2*^*−/−*^ cells had more significantly downregulated proteins, in keeping with CKS1 being a driver of protein degradation and CKS2 being important for stabilizing proteins.^25,26^ Gene ontology analysis demonstrated enrichment in general metabolic processes (including RNA processing) and interestingly proteins controlling gene expression and transcription in both *Cks1*^*−/−*^ and *Cks2*^*−/−*^ cells (Figure 3B–C). Relatively few differentially expressed proteins were conserved between *Cks1*^*−/−*^ and *Cks2*^*−/−*^ cells (Figure 3D), yet functional annotation of differentially abundant proteins revealed an enrichment in fundamental processes including transcription, apoptosis, protein translation, and key hematopoietic signaling pathways (Figure 3E, Suppl. Figure S4A).

When focusing on proteins which have been reported to be direct interactors of CKS1 and CKS2 (interactome-atlas.org), only 10 of the 21 proteins were detected in our dataset and of these, only 2 were differentially abundant. Both were CKS1 interactors, and both were significantly enriched in *Cks1*^*−/−*^ proteomes compared with *Cks2*^*−/−*^ and WT controls (Figure 3F–G). This indicated that the majority of functional differences in hematopoietic cells may be downstream of direct CKS1/2 interactions or through new partners. Therefore, we focused our analyses on differentially expressed proteins in our mass spectrometry analyses involving transcriptional control and noted these varied in their modality, with key hubs including Wnt signaling, MLL1/leukaemogenesis partners and the spliceosome predominantly differentially abundant in *Cks2*^*−/−*^ LSK cells (Figure 3H).

Transcription factors involved in MLL1/leukaemogenesis functions varied between WT, *Cks1*^*−/−*^, and *Cks2*^*−/−*^ LSK cells. The non-POU domain containing octamer binding protein (Nono) and promyelocytic leukemia protein were both significantly downregulated in *Cks1*^*−/−*^ cells compared with WT controls (Suppl. Figure S4B–C). In *Cks2*^*−/−*^, the ASH2 like histone lysine methyltransferase complex subunit (ASH2L) and elongation factor for RNA polymerase II (ELL) proteins were significant downregulated (Suppl. Figure S4D–E), and there are relatively few genes coregulated by these transcription factors (Suppl. Figure S4F).

The transcription factors involved in Wnt signaling were exclusively downregulated in *Cks2*^*−/−*^ cells, with the polymerase-associated factor (PAF1), mediator complex subunit 12 (Med12), and the PAF1 complex partner SH2 domain binding protein 1 (Ctr9), all significantly reduced in *Cks2*^*−/−*^ cells (Figure 3I–K). The gene targets of these 3 transcription factors overlap broadly (Figure 3L) and the general transcriptional regulatory targets across all 3 are predominantly Wnt signaling, oxidative stress, hypoxia inducible factor 1 alpha (Hif1α), and DNA damage (Figure 3M). Of the gene targets which overlap between all 3 proteins, there is a significant enrichment for Hif1α function, Wnt signaling, and the cell cycle (Figure 3N). We previously noted that regulation of the transcription factor MLL1 by the CKS1/CKS2 axis was important for regulating Wnt signaling in *Cks1*^*−/−*^ and *Cks2*^*−/−*^ mouse embryonic fibroblasts,^26^ and we confirmed a similar phenotype was present in hematopoietic cells from the same mice. *Cks1*^*−/−*^ LT-HSCs have lower active β-catenin compared with *Cks2*^*−/−*^ and WT controls (Figure 3O) and TCF/LEF activity is lower in cultured LSK cells from *Cks1*^*−/−*^ mice and higher in cultured LSK cells from *Cks2*^*−/−*^ mice compared with WT controls (Figure 3P).

### Transcriptomic analysis expands the regulatory network of CKS1 and CKS2 in HSPCs

**Figure 4.**
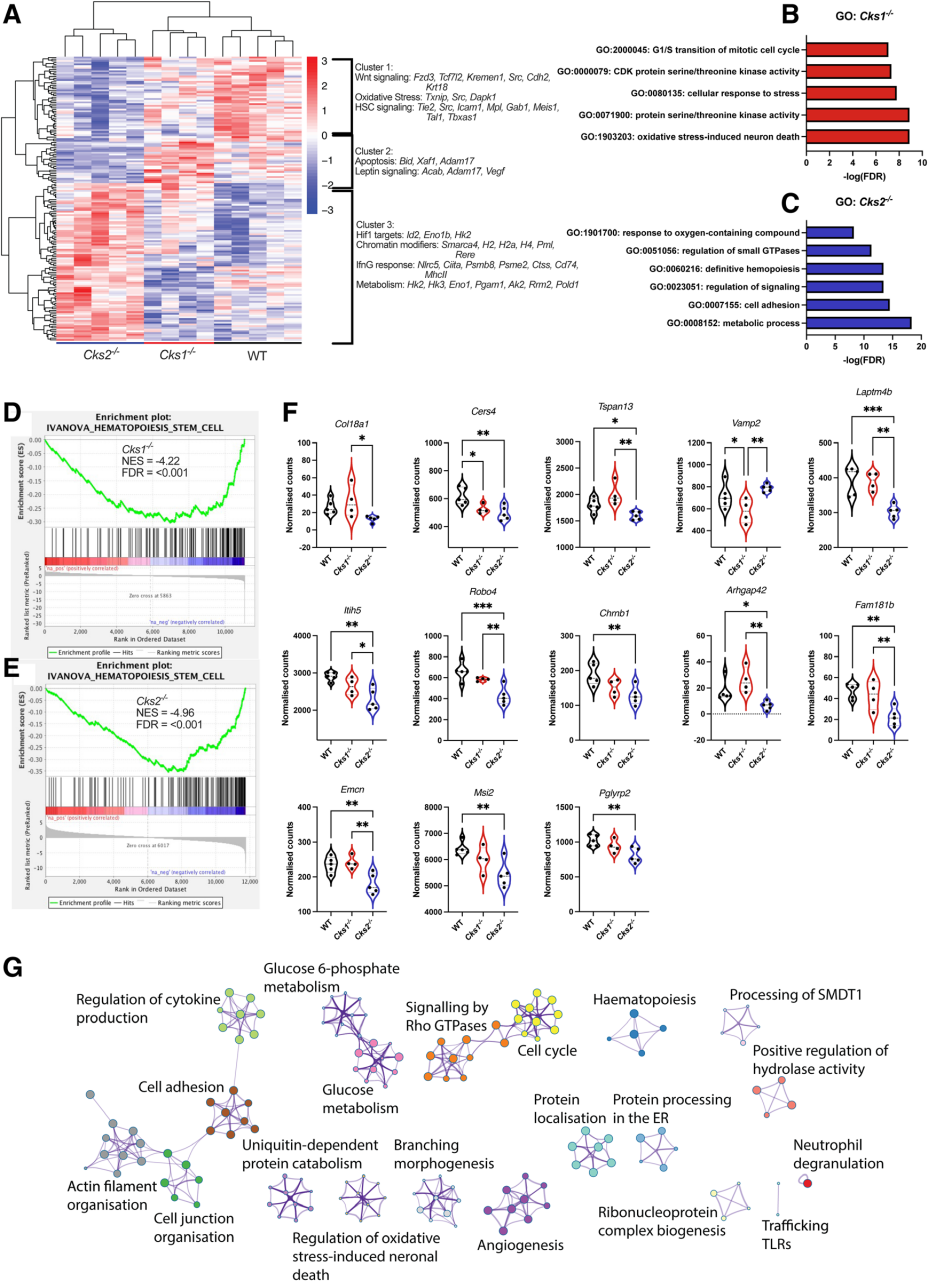
**The transcriptomic landscape of *Cks1*^−^/^−^ and *Cks2*^−^/^−^ hematopoietic stem and progenitor cells.** (A) Unsupervised clustering of differentially abundant genes between *Cks1/2*^*−/−*^ LSK cells and WT controls from RNA sequencing analysis. Gene ontology analysis of differentially abundant genes between (B) *Cks1*^*−/−*^ and (C) *Cks2*^*−/−*^ LSK cells vs WT controls. GSEA analysis of transcriptomic data from (D) *Cks1*^*−/−*^ and (E) *Cks2*^*−/−*^ LSK cells vs WT controls demonstrating downregulation of HSC signatures. (F) Individual differentially expressed genes from the Ivanova GSEA module. (G) Integrated analysis of transcriptomic and proteomic data using cytoscape.org. For all graphs a 1-way ANOVA was used to calculate significance of differences. *P < 0.05, **P < 0.005, ***P < 0.0005. ANOVA = analysis of variance; HSC = hematopoietic stem cell; LSK = lineage marker negative, Sca1^+^, cKit^+^; WT = wild type.

To further investigate changes in transcriptional control in *Cks1*^*−/−*^ and *Cks2*^*−/−*^ cells, we carried out whole transcriptome analysis by RNA sequencing of LSK cells. Interestingly, while both *Cks1*^*−/−*^ and *Cks2*^*−/−*^ transcriptomes are divergent to WT controls (Suppl. Figure S4G–H), there was no correlation between proteomes and transcriptomes (Suppl. Figure 4I). At the transcriptome level, *Cks2*^*−/−*^ LSK cells clustered further from WT controls than *Cks1*^*−/−*^ (Figure 4A), validating the increased number of altered transcriptional components in *Cks2*^*−/−*^ proteomes compared with *Cks1*^*−/−*^ (Figure 3E).

Unsupervised clustering of all genes differentially expressed in at least 1 comparison (WT versus *Cks1*^*−/−*^, WT versus *Cks2*^*−/−*^, and *Cks1*^*−/−*^ versus *Cks2*^*−/−*^) identified 3 clusters, each predominantly belonging to 1 genotype (Figure 4A). Cluster 1 (consisting of genes higher expressed in WT controls) contained Wnt signaling, oxidative stress, and definitive hematopoiesis genes. Cluster 2 (consisting of genes upregulated in *Cks1*^*−/−*^ cells) contained apoptosis and leptin signaling genes. Cluster 3 (consisting of genes upregulated in *Cks2*^*−/−*^ cells) contained Hif1 target genes, chromatin modifiers, Ifnγ response, and metabolism genes.

Gene ontology analysis suggests that *Cks1*^*−/−*^ cells are more engaged in protein modification and activity, whereas *Cks2*^*−/−*^ cells are more engaged in general hematopoietic processes (Figure 4B–C, respectively). Both *Cks1*^*−/−*^ and *Cks2*^*−/−*^ cells had altered metabolic and oxidative stress processes, mirroring what we had observed in proteomic profiles (Figure 3B–C). In agreement with functional phenotypes already noted, gene set enrichment analysis showed a significant reduction in transcriptomic signatures associated with HSC function in both knockout models (Figure 4D–E). Further analysis of individual genes from the Ivanova GSEA module revealed that 13 of these genes were differentially expressed (as opposed to the cumulative module being differentially enriched), with the majority downregulated in *Cks2*^*−/−*^ LSK cells and many differentially expressed between *Cks1*^*−/−*^ and *Cks2*^*−/−*^ (Figure 4F). Of those downregulated in *Cks2*^*−/−*^ critical genes such as the RNA binding protein *Musashi-2* (*Msi2*), the loss of which leads to poor LT-HSC transplantation^32^ and poor in vitro expansion of HSCs,^33^ and *Endomucin* (*Emcn*), which is a marker of repopulating HSCs,^34^ underline the important of CKS2 for regulating hematopoietic factors at the transcriptional level.

Integrated analysis of proteomic and transcriptomic data demonstrated that enriched proteins and transcripts are predominantly associated with protein modification, localization, and protein–protein interactions (Figure 4G). When focusing on cell cycle, signaling by Rho GTPases and metabolism nodes, there was an overlap between proteins and transcripts regulating Rho/Rac/Akt signaling and metabolism/oxidative stress. These results mirror our recent findings in primary human healthy and malignant hematopoietic cells, where CKS1-dependent protein degradation regulates signaling cascades controlling p27/Rac1/Akt and metabolic components.^21^ Together these data demonstrate that, despite the majority of individual protein changes being different, CKS1 and CKS2 regulate overlapping post-transcriptional cell signaling pathways in hematopoietic cells.

### The CKS1/CKS2 axis regulates protein networks critical for reducing HSC oxidative stress

**Figure 5.**
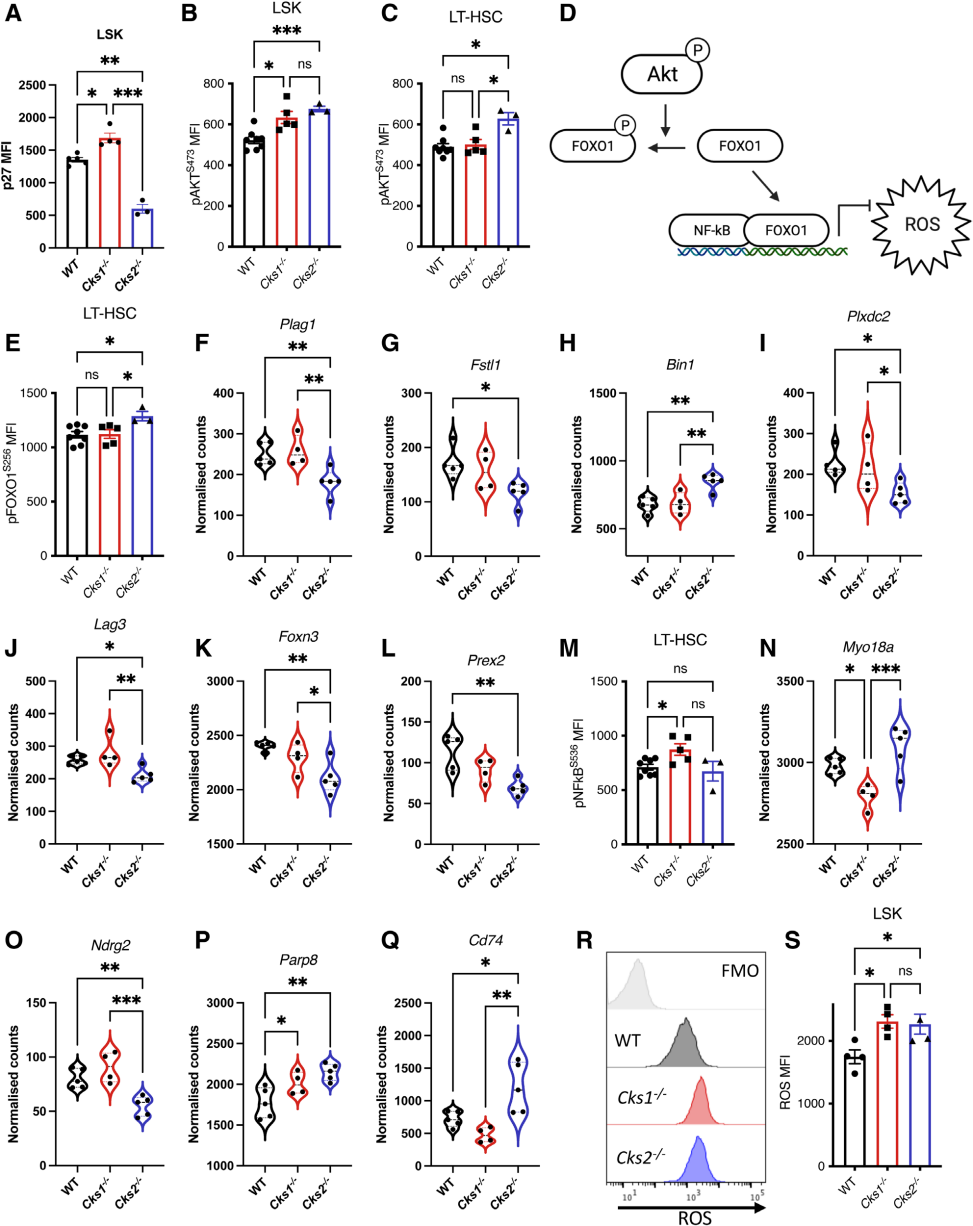
**Deficiency of *Cks1* or *Cks2* leads to unbalanced intracellular signaling pathway and ROS accumulation, resulting in poor LT-HSC function.** (A) MFI of p27 in LSK cells. MFI of Akt phosphorylated at Serine 473 in (B) LSK and (C) LT-HSC cells. (D) Diagram depicting Akt regulation of Foxo1 and Foxo1 interaction with NFκB for antioxidant gene expression. (E) MFI of Foxo1 phosphorylated at Serine 256 in LT-HSCs. (F–L) Individual differentially expressed genes from the Foxo1_01 GSEA module. (M) MFI of NFκB phosphorylated at Serine 536 in LT-HSCs. (N–Q). Individual differentially expressed genes from the NFKB_c GSEA module. (R) Flow cytometry histogram and (S) MFI of ROS in LSK cells. For all graphs, a 1-way ANOVA was used to calculate significance of differences. *P < 0.05, **P < 0.005, ***P < 0.0005. ANOVA = analysis of variance; LT-HSC = long-term hematopoietic stem cell; LSK = lineage marker negative, Sca1^+^, cKit^+^; MFI = mean fluorescence intensity; ROS = reactive oxygen species.

We and others have reported that CKS1 and CKS2 govern p27-dependent RhoA/Rac1/Akt activity in neuronal and leukemic cells.^21,35^ In agreement with these reports, in the hematopoietic system *Cks1*^*−/−*^ cells had increased p27 protein levels and *Cks2*^*−/−*^ cells had reduced p27 protein levels compared with WT controls (Figure 5A). CKS1/2-dependent regulation of the p27-Rho-Rac axis is important for maintaining balanced NADP/H metabolism in leukemic cells,^21^ and in *Cks1/2*^*−/−*^ LSKs cells, there is a significant unbalancing of NADP/H metabolism. Both *Cks1*^*−/−*^ and *Cks2*^*−/−*^ LSK cells have increased total NADP/NADPH levels compared with WT controls (Suppl. Figure S5A). This is predominantly due to an accumulation of the NADPH metabolite in both knockout lines (Suppl. Figure S5B). Although *Cks1*^*−/−*^ cells maintain a similar ratio of NADP/NADPH compared with WT controls, the strong increase in NADPH is partially at the expense of exhausting NADP in *Cks2*^*−/−*^ cells, leading to a significantly reduce NADP/NADPH ratio (or accumulation of the NADPH metabolite) in *Cks2*^*−/−*^ cells compared with WT controls (Suppl. Figure S5C).

Mechanistically, SKP2-CKS1/2-p27 and CDK-CKS1/2-p27 signaling converge on Akt activity^36^ and Rac1 and Akt have a reciprocal relationship.^37^ Considering Akt is a critical regulator of HSC function,^38,39^ we therefore investigated Akt status in *Cks1/2*^*−/−*^ hematopoietic cells. Both *Cks1*^*−/−*^ and *Cks2*^*−/−*^ LSK cells have higher Akt phosphorylation at Ser473 compared with WT controls (Figure 5B), a phenotype only conserved in *Cks2*^*−/−*^ LT-HSCs (Figure 5C). Interestingly, total Akt protein level is increased only in *Cks2*^*−/−*^ LSK and LT-HSCs, but not in *Cks1*^*−/−*^ cells compared with WT controls (Suppl. Figure S5D–E).

Together, CDK1/2, SCF^Skp2^, and pAkt^S473^ work in concert to phosphorylate Foxo1, an antioxidant transcriptional partner to NFκB (Figure 5D). In both *Cks1*^*−/−*^ and *Cks2*^*−/−*^ LSK and LT-HSCs, total Foxo1 protein levels are similar to WT control (Suppl. Figure S5F–G), but *Cks2*^*−/−*^ LSK and LT-HSCs had increased levels of Foxo1 phosphorylated at Ser256 (Figure 5E, Suppl. Figure S5H). Phosphorylation of Ser256 prevents Foxo1 reaching the nucleus for transcriptional functions, and in agreement, our transcriptomic analyses found reduced Foxo1-dependent gene transcription in *Cks2*^*−/−*^ LSK cells (Suppl. Figure S5I). Analysis of the individual genes comprising the Foxo1_01 GSEA module revealed 7 differentially expressed genes between *Cks2*^*−/−*^ LSK cells and WT controls (Figure 5G–L). Of these genes, the reduction in the zinc finger gene *Plag1* is of significant interest as it has recently been demonstrated that PLAG1 regulates *Msi2* expression (which is significantly downregulated at the RNA level in *Cks2*^*−/−*^ cells Figure 4F)^40^ and PLAG1 is a proteostatic regulator in HSCs itself, important for maintaining low protein synthesis rates in HSCs.^41^

The absence of a change in Foxo1 phosphorylation in *Cks1*^*−/−*^ cells compared with WT controls was surprising, but upon inspection of its key antioxidant transcriptional partner, NFκB, we found an analogous phenotype, with separation of total protein levels and phosphorylation status. Both *Cks1*^*−/−*^ and *Cks2*^*−/−*^ LT-HSCs had increased NFκB protein levels (Suppl. Figure S5J), but only *Cks1*^*−/−*^ LT-HSCs had increased NFκB phosphorylated at Ser536 (Figure 5M). This resulted in a similarly reduced NFκB-dependent transcriptional signature (Suppl. Figure S5K). Analysis of the individual genes comprising the NFKB_c GSEA module revealed 11 differentially expressed genes between either *Cks1*^*−/−*^ or *Cks2*^*−/−*^ LSK cells and WT controls (Figure 5N–Q, Suppl. Figure 5L–R).

The NADPH metabolite acts as a reservoir for Nox proteins to produce ROS, which can be balanced by increased antioxidant signaling.^21^ The increased levels of NADPH (Suppl. Figure S5B) and decreased antioxidant signaling (Figure 4E–Q) in *Cks1*^*−/−*^ and *Cks2*^*−/−*^ cells results in significantly increased ROS levels compared with WT controls (Figure 5R–S). The accumulation of intracellular ROS in HSCs is a well-documented cause of reduced LT-HSC functionality,^42^ demonstrating the requirement of CKS1 and CKS2 for maintaining low oxidative stress for correct HSC function.

## DISCUSSION

Studies of hematopoiesis and HSC biology have led the way in our understanding of stem cell biology and a deeper understanding of the functional proteome in HSCs has implications for regenerative medicine approaches (eg, bone marrow transplantation), bone marrow failure syndromes (eg, Diamond-Blackfan anemia), malignant transformation (eg, leukemia), and synthetic medicine approaches (eg, in vitro red blood cell or platelet production). Here, we demonstrate that balancing the CKS1/CKS2 axis is critical to maintain healthy HSC function and both CKS1 and CKS2 are required for successful HSC reconstitution.

In the absence of *Cks1* or *Cks2*, the composition of the hematopoietic system is substantially altered. Although there was a significant increase in the proportion of LSK and LT-HSCs in both *Cks1*^*−/−*^ and *Cks2*^*−/−*^ mice (Figure 1C and E), this did not result in improved functionality. Only *Cks2*^*−/−*^ bone marrow cells had higher clonogenic activity, whereas *Cks1*^*−/−*^ cells had reduced activity compared to control (Figure 1L). This was further confirmed in vivo, as both *Cks1*^*−/−*^ and *Cks2*^*−/−*^ LT-HSCs failed to robustly contribute to the hematopoietic reconstitution of recipient mice (Figure 2). The finding that both models have enrichment of LT-HSCs argues for a lack of differentiation and repopulation in competition with host bone marrow and carrier bone marrow. Additionally, while WT cells eventually exhaust through serial transplants, *Cks1*^*−/−*^ and *Cks2*^*−/−*^ were blocked in secondary transplants at the later MPP stage, again arguing for lower ability to progress through hematopoiesis upon transplant. CKS1 has been well documented to be a regulator of quiescence and G_1_/S phase transition, primarily through regulation of cell cycle inhibitors such as p27. In the hematopoietic system reduction of proliferation by *Cks1*^*−/−*^ LT-HSCs has been proposed as the underlying reason for poor colonization of recipient mice.^31^ CKS2 has opposing roles to CKS1 in p27 regulation and *Cks2*^*−/−*^ cells have a higher cell cycle velocity than WT controls.^25,26^ Interestingly, we demonstrated that *Cks2*^*−/−*^ HSPCs have an overlapping phenotype rather than an opposing phenotype to *Cks1*^*−/−*^ LT-HSCs. This would suggest that the main drivers of altered hematopoiesis are not solely dependent on p27 protein regulation and further study of the role of CKS1 and CKS2 in HSPCs may reveal new overlapping or independent phenotypes.

To investigate the underlying mechanism of altered functionality in the hematopoietic system in both *Cks1*^*−/−*^ and *Cks2*^*−/−*^ mice, we undertook an “omic” approach using mass spectrometry and RNA sequencing (Figures 3 and 4). It has recently been reported that the proteome and the transcriptome do not correlate in LT-HSCs, MPPs, and generally HSPCs^10^ and indeed we find a similar disconnect between the proteome and transcriptome when comparing alterations in *Cks1*^*−/−*^ *and Cks2*^*−/−*^ LSKs to WT controls (Suppl. Figure S4I). The proteomic profiles of *Cks1*^*−/−*^ and *Cks2*^*−/−*^ LSK cells compared with WT controls were largely independent at the individual protein level (Figure 3D) but overlapped more substantially in the specific biological processes functionally ascribed to each protein (Figure 3E). Where *Cks1*^*−/−*^ LSK cells had more significantly enriched proteins, *Cks2*^*−/−*^ LSK cells had more significantly depleted proteins (Figure 3A), a phenotype that would suggest the proteome levels are related to SKP2-CKS functions. Despite this, *Cks1*^*−/−*^ and *Cks2*^*−/−*^ models did not entirely phenocopy *Skp2*^*−/−*^ models, indeed *Skp2*^*−/−*^ mice have increased HSC populations and long-term reconstitution ability,^17,43^ a phenotype that would be conserved in *Cks1*^*−/−*^ and opposite in *Cks2*^*−/−*^ mice if their biological roles were solely through SKP2-dependent functions. The biological process with the most differentially abundant proteins across both genotypes was transcription and upon deeper analysis of transcriptionally active proteins, we found proteins involved in the Mll1 complex and Wnt signaling, both pathways known to be under the CKS1/CKS2 axis.^26^ Wnt signaling transcriptional regulators were significantly downregulated in *Cks2*^*−/−*^ LSK cells (Figure 3I–K), but TCF/LEF activity was increased in *Cks2*^*−/−*^ LSK cells grown in culture (Figure 3P). We previously found a similar mechanism in MEFs derived from *Cks2*^*−/−*^ mice, with reduced Mll1 protein levels and hyperactive Wnt signaling,^26^ and in agreement, Paf1 (which is a part of the Mll1 compass like complex^44,45^) is also downregulated in *Cks2*^*−/−*^ LSK cells at the protein level (Figure 3I). Wang et al^46^ previously demonstrated that *Skp2* is important for maintaining both β*-catenin* expression and expression of β-catenin target genes. This in turn was important to maintain HSC homing. In our findings, loss of CKS2 (which is competitive with CKS1 for SKP2 substrates) results in hyperactive Wnt signaling and a slight reduction in homing (Suppl. Fig. 2S), suggesting that unbalancing Wnt signaling, through the CKS1/CKS2 axis may not have identical effects to SKP2-dependent regulation. The regulation of transcription factor abundance at the protein level by CKS1 and CKS2 was surprising as previous reports indicated a role in RNA polymerase activity, DNA repair and chromatid separation, but not in direct transcription factor binding and activity.^25,29^ Only our previous report of altered Mll1 protein stability has demonstrated regulation of a transcription factor by CKS1 and CKS2.^26^ There has been consistent effort to alter transcription factor interactions with DNA in normal and malignant hematopoiesis, with little success in poor prognostic disorders, and indeed new degrader technology offers the most promise in malignancies such as AML.^47^ Therefore, the ability to control transcription factor protein levels by interfering with CKS1 and CKS2 activity may offer a new level of selectivity in proteostatic targeting.

To better understand the consequences of altered transcription factor abundance, we carried out paired RNA sequencing of LSK cells and found similarly divergent profiles between WT, *Cks1*^*−/−*^ and *Cks2*^*−/−*^ LSK cells (Figure 4A). *Cks2*^*−/−*^ LSKs had the most differentially abundant genes at the RNA levels. When we carried out an integrated analysis of all differentially abundant proteins or genes in both *Cks1*^*−/−*^ and *Cks2*^*−/−*^ LSKs compared with WT controls, we found a variety of signaling pathways related to the hematopoiesis, metabolism, oxidative stress, the cell cycle, and signaling by Rho GTPases (Figure 4G), which we chose to further investigate.

We previously reported that inhibition of CKS1-dependent protein degradation (and not CDK-associated functions) leads to the accumulation of p27 and an unbalance of both p27-RhoA-Rac1 signaling and p27-Akt signaling in healthy and malignant human hematopoietic cells.^21^ In HSPCs from *Cks1*^*−/−*^ and *Cks2*^*−/−*^ mice, we observed a significant increase of p27 in *Cks1*^*−/−*^ and a significant decrease of p27 in *Cks2*^*−/−*^ (Figure 5A). We further investigated the CKS1/2-Akt axis^21,36^ and found alterations in both protein levels and phosphorylation status. Akt activity was also changed, with the downstream target NFκB predominantly altered in *Cks1*^*−/−*^ LSK cells and Foxo1 predominantly altered in *Cks2*^*−/−*^ LSK cells compared with WT controls (Figure 5). Transcriptional profiles from both mouse models demonstrated that gene expression profiles from both Foxo1 and NFκB were altered (Figure 5, Suppl. Figure S5), demonstrating that while transcriptomes and proteomes did not correlate, we could find proteomic alterations under the influence of the CKS1/CKS2 axis that controlled critical hematopoietic transcriptomes.

Foxo1 and NFκB regulate anti-inflammatory and antioxidant transcriptional programmes, and together with the accumulation of metabolites important for the production of oxidative stress, alterations in these pathways in *Cks1*^*−/−*^ and *Cks2*^*−/−*^ LSK cells led to significantly higher intracellular ROS (Figure 5R-S). A decline in protein quality control, metabolism regulators and increase in intracellular ROS is well documented to result in poor LT-HSC functionality,^11,12,42,48^ demonstrating an underlying phenotype responsible for poor performance of *Cks1*^*−/−*^ and *Cks2*^*−/−*^ LT-HSCs after transplantation. These phenotypes point to CKS1 and CKS2 as actionable targets in bone marrow disorders where protein quality control and intracellular signaling declines or is hijacked by neoplastic mutations, and indeed we have already demonstrated that inhibition of CKS1 can be “chemoprotective” for healthy HSCs.^21^

Together these findings demonstrate that the fine balance of protein homeostasis, governed by the CKS1/CKS2 axis, is essential for LT-HSCs to overcome the stress of transplantation. Although the roles of epigenetics, transcription, and protein translation have been well documented in hematopoiesis, little is known about the functional state of the proteome in HSCs, and how the balance of protein modification and degradation maintains functional HSCs. Here we demonstrate that the small phospho-binding adaptor proteins CKS1 and CKS2 are essential for healthy HSC function during transplantation through a myriad of functions including intracellular protein signaling cascades, transcription factor abundance, and metabolic control. With the advent of low input proteomic approaches,^49^ functional kinome monitoring^18^ and improved proteomic predictive tools,^50^ the functional state of the proteome in HSCs will provide new insights into the biology of rare and highly specialized HSCs and offers much promise for engineering and regenerative medicine approaches.

## ACKNOWLEDGMENTS

We would like to acknowledge the Francis Crick core flow cytometry and biological research facilities, and Dr Maria Jassinskaja for support developing assays. We would like to thank Dr T. Milne and Dr J. Barlow for critical feedback on the manuscript. We would like to dedicate this manuscript to the late Dr Veronica Yu and Prof David Grimwade who supported WG early in his career, facilitating development of this project.

## AUTHOR CONTRIBUTIONS

WG conceived the study, designed and carried out experiments, analyzed data, and wrote the manuscript. SA and BR designed and carried out experiments and analyzed data. CH carried out experiments. PC carried out mass spectrometry analyses. MLS carried out RNA sequencing analyses. KB, DK, and PRC designed and supervised experiments. DB conceived and supervised the study and wrote the manuscript. All authors provided critical feedback on the manuscript pre-submission.

## DISCLOSURES

PRC is a co-founder and director of Kinomica Ltd. The D.G.K. lab receives research funding from STRM.bio. DK is a HemaSphere editor. All the other authors have no conflicts of interest to disclose.

## SOURCES OF FUNDING

This work was supported partly by Cancer Research UK (FC001015 to DB), the UK Medical Research Council (FC001015 to DB), the Wellcome Trust (FC001015 to DB), a CRUK program grant (C15966/A24375 to DB), Leukaemia U.K. (2021/JGF/002 to WG), The Children’s Cancer and Leukaemia Group (LPT2021B44 to WG), and The Lady Tata Trust (to WG). The D.G.K. laboratory is supported by a Blood Cancer U.K. Bennett Fellowship (15008), an ERC Starting Grant (ERC-2016-STG-715371), an MRC-AMED joint award (MR/V005502/1), the MRC National Mouse Genetics Network, and a Cancer Research U.K. Programme Foundation Award (DCRPGF\100008). The K.S.B. laboratory is supported by a Kay Kendall Leukaemia Fund Intermediate Fellowship and a Wellcome Trust ISSF/University of York Centre for Future Health Fellowship.

## DATA AND MATERIALS AVAILABILITY

All data associated with this study are present in the paper or Suppl. materials. The mass spectrometry proteomics data have been deposited to the ProteomeXchange Consortium via the PRIDE partner repository (PXD035984) and the RNA sequencing data have been deposited to GEO.

## Supplementary Material


