# Supplementary material for: The CKS1/CKS2 Proteostasis Axis Is Crucial to Maintain Hematopoietic Stem Cell Function
Source: Hemasphere. 2023 Feb 28;7(3):e853. doi: 10.1097/HS9.0000000000000853 (PMC9977483; doi:10.1097/HS9.0000000000000853)
Supplement: Supplementary file 1 [file hs9-7-e853-s001.docx]

Supplementary Information

Supplementary methods

Proteomic analysis – Mass spectrometry

For proteomic analysis of LSK cells were freshly isolated from bone marrow of WT and *Cks1^-/-^* and *Cks2^-/-^* mice. 25,000 LSK cells were sorted from each mouse and samples were pooled with 3 donors per replicate per genotype for each n in the mass spec run. Pellets of LSK cells were lysed in 100 µL of urea buffer (8 M urea in 20 mM HEPES, pH: 8.0), lysates were further homogenized by sonication (30 cycles of 30s on 30s off; Diagenode Bioruptor Plus) and insoluble material was removed by centrifugation. Protein amount was quantified using BCA (Thermo Fisher Scientific). Samples were pooled 2 in 1 and diluted in urea buffer to obtain 4 µg of protein in a volume of 200 µL. Therefore, each replicate sample originates from 2 independent mice and can be considered as completely independent biological replicates. Protein samples were subjected to cysteine alkylation by sequential incubation, at 25°C and agitation, with 10 mM dithiothreitol (DDT) for 1h and 16.6 mM iodoacetamide (IAM) for 30 min. Then, 600 µL of 20 mM HEPES (pH 8.0) were added to the protein suspensions to reduce the urea concentration to 2 M. Trypsin beads (50% slurry of TLCK trypsin; Thermo-Fisher Scientific; Cat. #20230) were equilibrated with 3 washes with 20 mM HEPES (pH 8.0) and 100 µL of equilibrated trypsin beads were added to the protein suspensions. Proteins were digested overnight at 37°C and trypsin beads were removed by centrifugation (2000 xg at 5°C for 5 min). The resulting peptide solutions were desalted using carbon C18 spin tips (Glygen; Cat. #TT2MC18). Briefly, spin tips were activated and equilibrated twice with 200 µL of Elution Solution (70% ACN, 0.1% TFA) and Wash Solution (1% ACN, 0.1% TFA), respectively. Samples were loaded and spin tips were washed twice with 200 µL of Wash Solution. For peptide elution, 50 µl of Elution Solution was added 4 times to the spin tips. In each desalting step, spin tips were centrifuged at 1,500xg at 5°C for 3 min. Finally, eluted peptides were dried in a SpeedVac and peptide pellets were stored at −80°C.

Proteins in each sample were identified and quantified using Mass spectrometry as previously described^20^. Briefly, peptide pellets were resuspended in 3 µL of reconstitution buffer (20 fmol/µL enolase in 3% ACN, 0.1% TFA), and 2 µL were loaded onto an LC-MS/MS system consisting of a Dionex UltiMate 3000 RSLC coupled to a Q Exactive Plus Orbitrap Mass Spectrometer (Thermo Fisher Scientific) through an EASY-Spray source (Cat. # ES081, Thermo Fisher Scientific). Mobile phases for the chromatographic separation of the peptides consisted in Solvent A (3% ACN: 0.1% FA) and Solvent B (99.9% ACN; 0.1% FA). Peptides were loaded in a micro-precolumn (Acclaim PepMap 100 C18 LC; Cat. # 160454, Thermo Fisher Scientific) and separated in an analytical column (Acclaim PepMap 100 C18 LC; Cat. # 164569, Thermo Fisher Scientific) using a gradient running from 3% to 23% over 120 min. The UPLC system delivered a flow of 2 µL/min (loading) and 300 nL/min (gradient elution). The Q-Exactive Plus operated a duty cycle of 2.1s. Thus, it acquired full scan survey spectra (m/z 375–1500) with a 70,000 FWHM resolution followed by data-dependent acquisition in which the 15 most intense ions were selected for HCD (higher energy collisional dissociation) and MS/MS scanning (200–2000 m/z) with a resolution of 17,500 FWHM. A dynamic exclusion period of 30s was enabled with a m/z window of ±10 ppms.

Peptide identification from MS data was automated using the Mascot Daemon (v2.6.1) platform in which Mascot Distiller (v2.5.1) generated peak list files (MGFs) from RAW data and the Mascot search engine matched the MS/MS data stored in the MGF files to peptides using the SwissProt Database restricted to mouse taxon (SwissProt_2016Oct.fasta; 16,838 sequences). Searches had a FDR of ~1% and allowed 2 trypsin missed cleavages, mass tolerance of ±10 ppm for the MS scans and ±25 mmu for the MS/MS scans, carbamidomethyl Cys as a fixed modification and PyroGlu on N-terminal Gln and oxidation of Met as variable modifications. Identified peptides were quantified using Pescal software in a label free procedure based on extracted ion chromatograms (XICs). Thus, the software constructed XICs for all the peptides identified across all samples with mass and retention time windows of ±7 ppm and ±2 min, respectively and calculated the area under the peak. Individual peptide intensity values in each sample were normalized to the sum of the intensity values of all the peptides quantified in that sample. Data points not quantified were given a peptide intensity value equal to the minimum intensity value quantified in the sample divided by 10. Protein intensity values were calculated by adding the individual normalized intensities of all the peptides comprised in a protein, and protein score values were expressed as the maximum Mascot protein score value obtained across samples.

Transcriptomic analysis – RNA sequencing

Read adaptor removal and trimming was carried out with Trimmomatic (0.36)[1]. Read alignment to the mouse GRCm38 release 89 was performed with STAR( v2.5.2a)[2] and gene level quantification was done with RSEM ( v1.2.31)[3]. Gene expression counts per gene and per sample were imported into DESeq2 (v1.20.0)[4] where Differential expression analysis was carried out. A gene was considered differentially expressed if it had a p adjusted value < 0.05. For Gene Set enrichment analysis, GSEAPreranked analysis was carried out in GSEA [5], using ranked files that were prepared by sorting differentially expressed genes by the wald statistic from DESEq2, and fed into GSEA GUI (v2.2.3). All parameters were kept as default except we used "classic" as the enrichment statistic and we allowed larger data sets to be up to 500000. All plotting was carried out in an R environment (v3.4.1)[6] using ggplot2 [7].

TOPFlash assay

LSK cells were co-transfected with 5 ug M50 Super 8x TOPFlash (#12456; Addgene) or control M51 Super 8x FOPFlash (#12457; Addgene) and 100ng Renillar control (pRL-TK; Promega). Luciferase activity was measured using the dual luciferase assay system (Promega). Relative luciferase activity (RLA) was calculated as Luciferase/Renilla signal.

Additional References:

[1] Trimmomatic: a flexible trimmer for Illumina sequence data.. Bolger AM, Lohse M, Usadel B. Bioinformatics. 2014 Aug 1;30(15):2114-20.

[2] Dobin A, Davis CA, Schlesinger F, et al. STAR: ultrafast universal RNA-seq aligner. Bioinformatics. 2012;29(1):15-21.

[3] RSEM: accurate transcript quantification from RNA-Seq data with or without a reference genome. Li B, Dewey CN. BMC Bioinformatics. 2011 Aug 4;12:323. doi: 10.1186/1471-2105-12-323.

[4] Michael I Love, Wolfgang Huber and Simon Anders (2014): Moderated estimation of fold change and dispersion for RNA-Seq data with DESeq2. Genome Biology

[5] Subramanian, Tamayo, et al. (2005, PNAS) and Mootha, Lindgren, et al. (2003, Nature Genetics).

[6] (https://www.r-project.org/)

[7] Wickham H (2016). ggplot2: Elegant Graphics for Data Analysis. Springer-Verlag New York. ISBN 978-3-319-24277-4

Key resources table

| **Reagent/Resource** | **Source** | **Identifier** |
| --- | --- | --- |
| **Antibodies** | | |
| Lineage cocktail | BD biosciences | 340546 |
| c-Kit (CD117) | BD biosciences | 2B8 |
| Sca-1 | BD biosciences | D7 |
| CD48 | Biolegend | HM48-1 |
| CD150 | Biolegend | TC15-12F12.2 |
| CD34 | BD biosciences | RAM34 |
| Flt3 (CD135) | Biolegend | A2F10 |
| CD16/32 | Biolegend | 93 |
| CD127 | Biolegend | A7R34 |
| CD45.1 | Biolegend | A20 |
| CD45.2 | Biolegend | 104 |
| Gr-1 | ebioscience | RB6-8C5 |
| Mac1 (CD11b) | BD biosciences | M1/70 |
| CD4 | BD biosciences | H129 |
| CD8 | BD biosciences | 53-6.7 |
| B220 | ebioscience | RA3-6B2 |
| Non-p-β-catenin | CST | D2U8Y |
| p27 | CST | D69C12 |
| AKT | CST | C67E7 |
| pAKT(S473) | CST | D9E |
| Foxo1 | CST | C29H4 |
| pFoxo1(S256) | Antibodies Online | ABIN684755 |
| NFκB | CST | D14E12 |
| pNFκB(S536) | CST | 93H1 |
| **Probes & Chemicals** | | |
| CellRox DeepRed | ThermoFisher | C10422 |
| N-acetyle-L-cysteine (NAC) | Sigma | A7250 |
| **Mouse models** | | |
| *Cks1^-/-^* | Frontini et al. (2012) | Cks1bGt(S8-1h1)Sor |
| *Cks2^-/-^* | Frontini et al. (2012) | Cks2Gt(D027C12)Wrst |
| **Software** | | |
| R software v3.6.1 | R project | r-project.org |
| Flowjo v10.6.1 | Flowjo | N/A |
| Prism 7 | GraphPad | N/A |

**GSEA modules**

IVANOVA_HEMATOPOIESIS_STEM_CELL

MAPPED_SYMBOLS: JUN,NFIX,LAPTM4B,CDIP1,ZBTB37,MYO10,GATA3,PKD2,COL4A1,IFT81,NR4A1,SMARCA2,USP34,INPPL1,POLR2A,PRKCE,IFIH1,DDIT4,SAMD10,CPXM1,SLC41A1,PEX11A,REST,SASH1,ASH1L,ZRSR2,NFKBIA,CPSF2,ITGA3,MEF2D,SLC23A2,,ITIH5,RFPL4B,SGSM1,PLAC8L1,,NFATC2,PITPNC1,VLDLR,MARCHF9,PRCD,,FAM181B,ZDBF2,,,TCF25,PARD6G,HOXB2,IGDCC4,MSI2,NFKBIE,ROBO4,HOOK1,,SMC5,EFNA3,ABCC12,,HYAL1,PEAK1,ZBTB20,ARHGAP42,,PALMD,SLC16A5,PAIP1,ITGA9,MIRLET7D,CYP2D6,,COBLL1,ETV3,,MYO10,NKAIN3,,PGLYRP2,,NCOA2,SCAF11,RILPL1,CHN1,,IFI44,PPP1R9A,DNMT3A,PDE4B,,SEMA3D,ZC2HC1A,GNL1,NTRK3,LPAR4,NRXN1,SREK1,,ESR1,,ZBTB20,SERPINB8,,KCNK15,PTGR1,MSI2,ENG,DNAJB13,KLHL26,,LYPD1,CD164L2,FAM168B,RPS6KA3,,ARHGAP32,HNRNPR,CLPS,TRIM61,PCK1,TCF4,DEF8,,USP38,FRMD8,GATAD2A,PYGM,PTPRK,,ZCCHC7,SOS1,GAPDHS,SYN2,IQGAP2,SEC14L3,FAM110B,TCTN2,SV2A,KIF5A,IL36A,VPS37A,SEMA4C,SLIT2,SOCS5,SLC25A30,SEPTIN3,BAALC,NR4A2,PRKG1,RBM28,DUSP5,,ZXDB,,CERS4,NSD3,MAFF,SPTY2D1OS,TRIM9,SULT4A1,LRRC49,CTNNAL1,CAMTA1,FUNDC2,DYDC1,,CCDC136,CCDC185,SLC35F1,FBXW12,,,,NRXN3,WNT6,,MEX3A,ITIH5,SYT11,LRRTM4,ARL4C,SMAD7,,KCNJ6,GEM,TSPAN6,TBXA2R,ARHGEF28,ETV1,NFATC2,PRKACB,CDK14,MYCBP2,DNAJC21,AGXT,CHRNB1,WWC2,CYP2E1,PDC,GLB1L,FOXB2,SKIL,EMCN,CCAR1,TSPAN13,RBX1,,S100A5,,JADE1,NKX28,VLDLR,CCL19,CXCL14,CHRNA6,THA1P,ZC3H12C,EMP1,SIAH2,ZMYND8,PDF,EGR1,VAMP2,RASD1,TGOLN2,BCL2L11,COL18A1,RBBP6

FOXO1_01

MAPPED_SYMBOLS

RBFOX1,ABLIM3,AKT1S1,ANKRD28,AP4M1,APOA2,ARID4A,ASB7,ASXL1,ATF7IP,AXIN2,BCL11B,BDNF,PHF21A,ARFGEF1,BIN1,BMF,BMI1,BUB3,WBP1L,C18orf25,C1QTNF3,FAM110A,WDR46,OARD1,NPVF,CADPS,,CBFA2T2,CCND1,CCNG2,CDKN1C,CHCHD7,CHD1,FOXN3,KCNJ5,CLDN2,CNNM3,CRB1,CREM,CRH,CSAD,CSNK1G3,CXXC5,CCN1,DDIT4,DDX17,DEDD,PREX2,WIPI2,SLC10A7,DLX1,DMD,DTNA,DUSP1,EFEMP1,EIF4EBP2,ELAVL2,ELOVL6,EML1,EMP1,EPHB1,EPHB3,ESRRG,ETV1,ETV5,EXT1,EZH1,FGF12,FGF14,FGF9,HMCN1,GPR174,ZNF654,ZMAT4,PRPF38A,,PTCHD1,C12orf50,CCDC89,C2orf69,PTCHD4,LINC00173,,FOXO1,FOXP2,FST,FSTL1,GATA6,GFRA1,HCAR2,HCAR3,ADGRF1,GPR18,GPR63,GPRC5C,GRB7,GRIK3,GTF2B,H2AZ2,H2AX,H33B,HBP1,HESX1,HHEX,HIPK1,HTN1,PFDN6,NSG2,HOXA11,HOXA3,HOXB4,HOXB8,HOXC4,HSPB2,IGSF1,IL25,IL1RAPL1,IMPDH2,INHBA,IRS4,ITPR3,KDM3A,KLHL40,KCNJ15,KCNN1,FAM53B,RTL9,KLF12,KLHDC2,KRT85,LAG3,LASP1,LCP2,LHX1,LHX5,LMO3,LMO4,SZT2,CREBRF,SIAH3,SLC25A34,IER5L,TMEM88,ADGRL2,LRRC1,MCM7,MED8,MXRA8,ATXN7L1,,NIM1K,ODF3L1,MGLL,MITF,KMT2E,NDRG1,NF1,NFIB,NFIX,NFYA,C1orf43,NKX22,NMNAT2,NNT,NR2C2,NR4A2,NRG1,NTRK3,TPD52L3,LUC7L3,OLIG3,OPA3,ORC1,OSR1,PALM,PAPPA,PDCD4,PHACTR3,PITX2,PLAG1,PLCB2,PLXDC2,PMCH,POU3F2,POU3F4,PPP1CB,PRDM1,PROX1,PTPRD,PURA,RASGEF1B,TRIM63,RUNX1,SALL3,SCML1,SCRN3,SCUBE3,SDK2,TSPYL2,SEMA4G,SEMA6D,SESN3,SREK1,SH3BP5,SH3GL3,SLC25A28,SLC26A2,SLC26A7,SLC38A2,SLITRK5,SLITRK6,SMAD1,SNCAIP,SOX2,SPAG9,SSBP3,STOML3,SYT16,SYT6,CSRNP3,TAS2R39,TAFAZZIN,TBC1D17,TBCC,TBX4,ELOA2,TDRD5,THRA,CRTC2,TRIM8,TSC1,UBE2H,UBL3,NRN1L,USP3,KDM6A,WBP4,WFIKKN2,NSD3,LINS1,YTHDF3,OTUD7B,ZADH2,ZEB2,ZFYVE1,ZBTB16,ZBTB18,ZBTB22,ZNF366,ZNF385B,IKZF2

NFKB_c

MAPPED_SYMBOLS

ACAN,AKR1B1,ALG6,AMOTL1,ANKHD1,ANP32A,APPL1,ARHGAP5,ARHGAP8,ARHGEF2,ARPC5,ASCL3,ASH1L,BAHD1,ADGRB2,BCKDK,BCL11A,BCL2L1,BCL3,BDNF,BHLHE40,CXCR5,BMF,MIR17HG,MIDEAS,C1QL1,PHF20,RFTN2,CA9,NOS1AP,CARTPT,CCDC8,CCL22,CCL5,CD247,CD74,CDC14A,CDC37,CDH5,CDK6,ACAP3,CGGBP1,CHD4,VSX2,CLCN1,COL11A2,CTAGE4,CCN2,CUEDC1,CXCL10,TASL,CYB5A,CYLD,DACH2,DAP3,DCLK1,DDR1,DDX17,GPBP1,DNAJA1,DUSP22,EBF1,ECT2,S1PR2,EFNA3,EGF,EGR2,EGR3,EHF,EIF5A,ENO3,EPHB6,FGF1,FGF17,FLI1,KANSL3,SOBP,INO80D,PARP8,PTCHD1,FAM241A,LINC01138,FOS,FOXA3,FOXD2,FOXD3,FOXJ2,FTHL17,FUT7,G3BP1,GABPB2,GADD45B,GATA4,GNA13,GNAO1,GNG4,GPHN,GRIN2D,GRK5,RSF1,HCFC1,HOXA11,HOXB6,HOXB9,HSD3B7,ICAM1,ID2,IER5,IFNB1,IL13,IL18R1,IL1A,IL1RAPL1,IL1RN,IL2,IL23A,IL27,IL27RA,IL2RA,IL4I1,IRF1,ITGB4,ITGB8,JAK3,JPH3,KCNQ5,ARHGAP44,HHATL,,LAMB3,LASP1,LCAT,LCN2,TINAGL1,LIN28A,SH2B3,NOTUM,ZNF436AS1,ZNF800,ZNF821,FAM117A,TMEM88,LRCH1,LRRFIP2,LTB,LYPLA2,MAG,MAML2,MAP3K13,MAP3K8,ANKHD1EIF4EBP3,ALKBH6,CCDC107,LIX1L,MLLT6,MNT,MOB3C,PNKD,MRPS6,MSC,MSX1,NDRG2,NFAT5,NFKB2,NFKBIA,NFKBIB,NLK,NOS2,NUP153,NPAS4,LUC7L3,OPCML,PAPPA,PCBP4,PCSK2,PFN1,PHF6,PIGC,PLA1A,PLP1,GDPD5,PPP1R13B,PPP2R5E,PPP3CA,PRKCD,AAMDC,PTGES,PTGS2,PTHLH,PTMS,PTPRJ,PURG,,RAB10,RAD18,RALGDS,RANBP10,RAP2C,RGL1,RHOA,RHOG,RIN2,RND1,RORC,S100A10,SCRT2,SEC14L2,SEMA3B,SESN2,SCAF4,SIN3A,SIRT2,SIX5,SLAMF8,SLC34A3,SLC43A1,SLC6A12,KCNT2,SMARCAD1,SMOC1,SMPD3,SNAP25,SNX15,SOCS2,SOX10,SOX3,SOX5,SP6,SPTB,STAT6,STX4,SYMPK,TAL1,TAFAZZIN,TCEA2,TCTA,TFE3,MYO18A,TJAP1,CD40,TNFSF15,TNFSF18,CD70,TNIP1,TNIP3,TNR,TOP1,TP63,HSP90B1,TRIB2,TRIM47,TSLP,TSNAXIP1,TYRO3,UACA,UBE2I,SUN2,PAN2,VCAM1,VNN3P,WNT10A,WRN,XPO6,YY1AP1,ZADH2,ZHX2,VEZF1,ZNF217,ZNF384,ZNF436

Supplementary figures


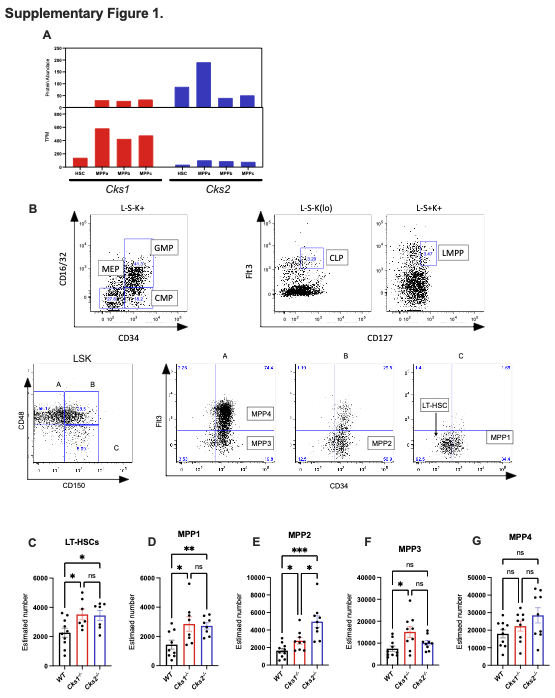


**Supplementary Figure S1. A.** Protein and RNA abundance of Cks1 and Cks2 LT-HSCs and MPPs from Zaro et al. (2020)^10^. **B.** Example gating strategy for immunophenotypic populations. Note above each plot is the parent gate leading to that plot. Estimated number of **C.** LT-HSCs, **D.**  MPP1, **E.**  MPP2, **F.**  MPP3 and **G.** MPP4 cells per mouse based on total bone marrow cellularity and percentage frequency of individual cell types. For all graphs a one-way anova was used to calculate significance of differences. *p<0.05, **p<0.005, ***p<0.0005.


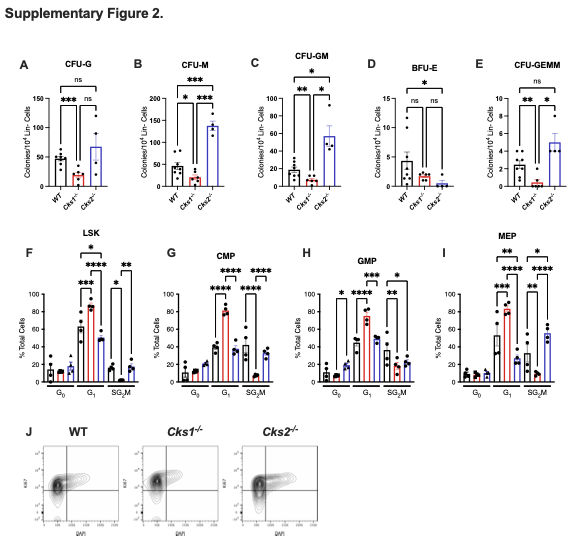


**Supplementary Figure S2.** Colony forming units **A.** Granulocyte, **B.** Macrophage, **C.** Granulocyte/Macrophage, **D.** Burst forming unit erythroid, **E.** Granuloctye, erythrocyte, macrophage, megakaryocyte, per 10^4^ lineage marker negative bone marrow cells. Cell cycle analysis of **F.** LSK, **G.** CMP, **H.** GMP and **I.** MEP populations, with **J.** an example gating strategy from WT and *Cks1^-/-^* and *Cks2^-/-^* mouse bone marrow cells. For all graphs a one-way anova was used to calculate significance of differences. *p<0.05, **p<0.005, ***p<0.0005.

**Supplementary Figure S3. A.** Percentage donor derived CD45.2 cells of total CD45 positive cells in the spleen of primary recipient mice. Percentage **B.** MPP2, **C.** MPP3, **D.** MPP4, **E.** CMP, **F,** GMP, **G.** MEP, **H.** LMPP and **I.** CLP cells of total CD45.2 donor derived cells in primary recipient bone marrows. **J.** Breakdown of colony forming units per 10^4^ lineage negative CD45.2 positive cells seeded from primary recipients. **K.** Percentage of donor derived CD45.2 cells of total CD45 positive cells in the spleen of secondary recipient mice. Percentage  **L.** LT-HSC, **M.**  CMP,  **N.** GMP,  **O.**  MEP,  **P.**  LMPP and  **Q.** CLP cells of total CD45.2 donor derived cells in secondary recipient bone marrows. **R.**  Breakdown of colony forming units per 10^4^ lineage negative CD45.2 positive cells seeded from secondary recipients. **S.** Analysis of CD45.2 positive cells in the bone marrow of WT CD45.1 recipients 16 hours after injection of WT, *Cks1^-/-^* and *Cks2^-/-^* LSK cells. **T.** Peripheral blood chimerism of CD45.2 donor LT-HSCs weeks after intra-bone injection. For all graphs a one-way anova was used to calculate significance of differences. *p<0.05, **p<0.005, ***p<0.0005. ****p<0.0001.

**
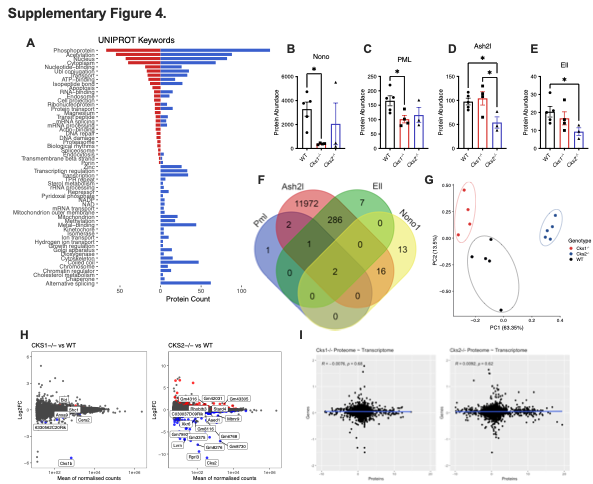
**

**Supplementary Figure S4. A.** Uniprot key words for differentially abundant proteins between *Cks1^-/-^* (Red) or *Cks2^-/-^* (Blue) and WT LSK cells. Protein abundance of the key transcription factors **B.**  Nono,  **C.** PML,  **D.**  Ash2l and **E.** Ell in *Cks1/2^-/-^* LSK cells vs WT controls. **F.** Venn diagram depicting overlap of transcriptional targets of the indicated transcription factors from Chip-atlas.org. **G.** PCA plot for transcriptomic data from WT and *Cks1/2^-/-^* LSK cells. **H.** Log2 fold change vs mean of normalised counts for transcriptomic analysis of *Cks1/2^-/-^* vs WT controls. **I.** Correlation of log2 fold change values from transcriptomic and proteomic data sets for *Cks1/2^-/-^* vs WT controls. For all graphs a one-way anova was used to calculate significance of differences. *p<0.05, **p<0.005, ***p<0.0005. ****p<0.0001.

**Supplementary Figure S5. A.** Total NADP/NADPH and **B.** NADPH abundance, and **C.** NADP/NADPH ratio in *Cks1/2^-/-^* and WT LSK cells. Mean fluorescence intensity (MFI) of Akt in **D.** LSK and **E.**  LT-HSC cells from WT and *Cks1/2^-/-^* mice. MFI of Foxo1 in **F.** LSK and **G.**  LT-HSC cells from WT and *Cks1/2^-/-^* mice. **H.** MFI of phosphorylated Foxo1 at Serine 256 in LSK cells from WT and *Cks1/2^-/-^* mice. **I.** Foxo1 GSEA score in *Cks2^-/-^* vs WT LSK transcriptomes. **J.** MFI of NFκB in LSK cells from WT and *Cks1/2^-/-^* mice. **K.** NFκB GSEA score in *Cks1^-/-^* vs WT LSK transcriptomes. **L-R.** Individual differentially expressed genes from the NFKB_c GSEA module. For all graphs a one-way anova was used to calculate significance of differences. *p<0.05, **p<0.005, ***p<0.0005.
