# Supplementary figures and images for: The CKS1/CKS2 Proteostasis Axis Is Crucial to Maintain Hematopoietic Stem Cell Function
Source: Hemasphere. 2023 Feb 28;7(3):e853. doi: 10.1097/HS9.0000000000000853 (PMC9977483; doi:10.1097/HS9.0000000000000853)

Supplementary Figure 1.

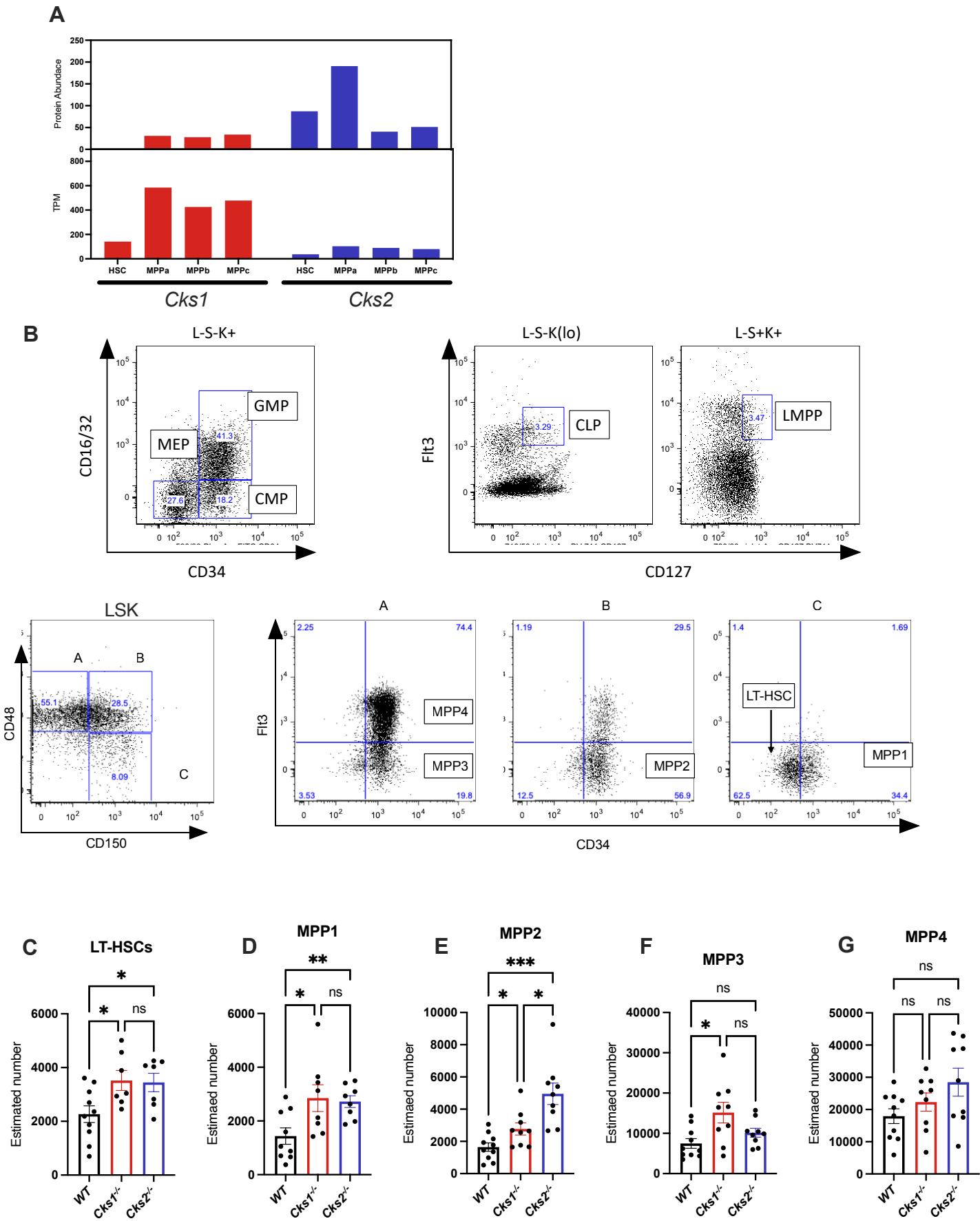

Supplement: Supplementary file 2 [file hs9-7-e853-s002.pdf]

Supplementary Figure 2.

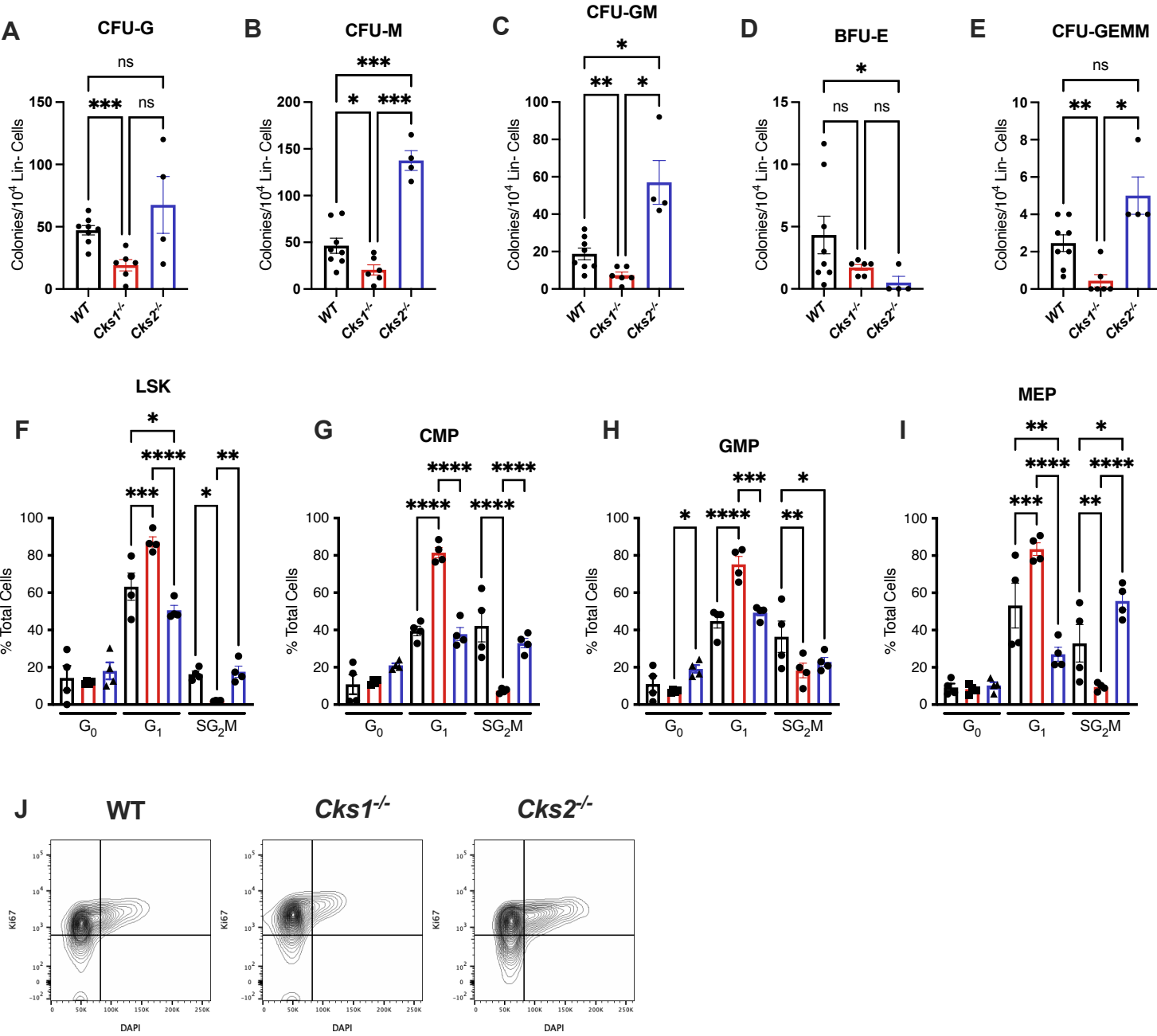

Supplement: Supplementary file 3 [file hs9-7-e853-s003.pdf]

Supplementary Figure 3.

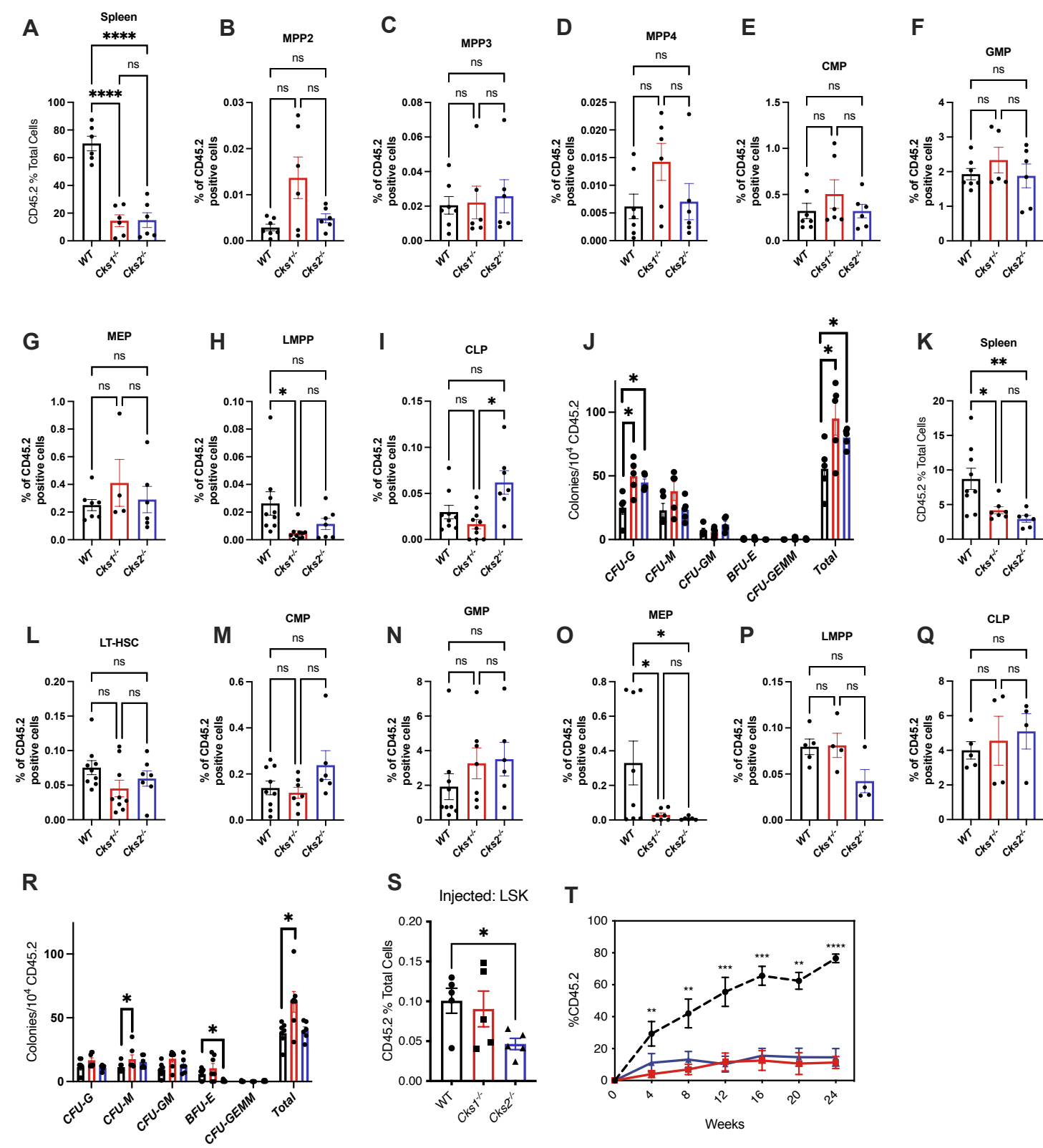

Supplement: Supplementary file 4 [file hs9-7-e853-s004.pdf]

Supplementary Figure 4.

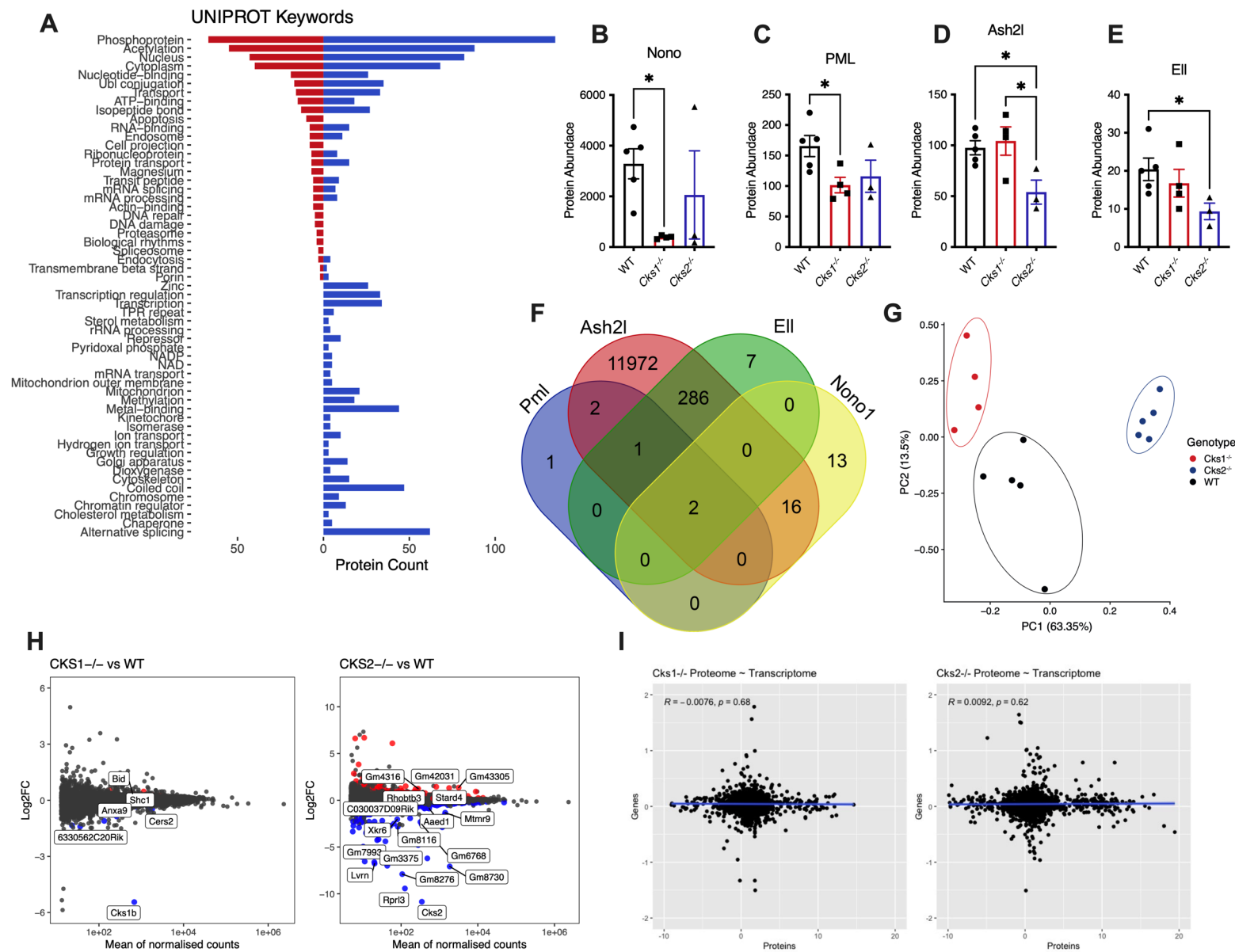

Supplement: Supplementary file 5 [file hs9-7-e853-s005.pdf]

Supplementary Figure 5.

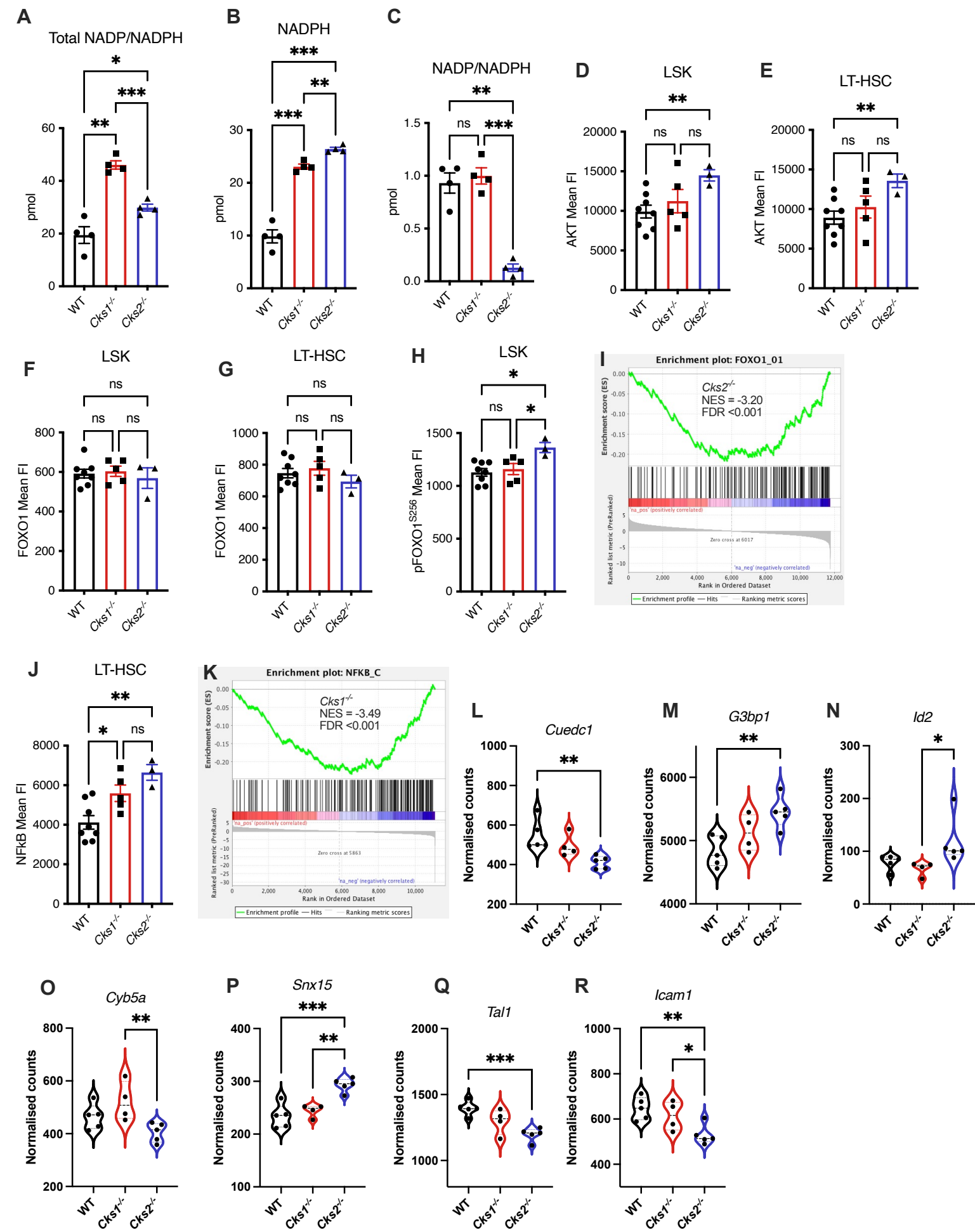

Supplement: Supplementary file 6 [file hs9-7-e853-s006.pdf]
